# The good life in rural and urban Senegal: A qualitative and quantitative study

**DOI:** 10.1371/journal.pone.0252134

**Published:** 2021-05-27

**Authors:** Priscilla Duboz, Enguerran Macia, Amadou H. Diallo, Emmanuel Cohen, Audrey Bergouignan, Sidy M. Seck

**Affiliations:** 1 IRL 3189 *Environnement*, *Santé*, *Sociétés*, Faculté de médecine, CNRS / Université de Bamako / CNRST Burkina-Faso, Université Cheikh Anta Diop, Dakar, Senegal; 2 UMR 7206 *Eco-Anthropologie*, CNRS / MNHN / Université de Paris, Site du Musée de l’Hommes, Paris, France; 3 UMR 7178 Institut Pluridisciplinaire Hubert Curien, CNRS / Université de Strasbourg, Strasbourg, France; 4 Division of Endocrinology, Metabolism and Diabetes, Anschutz Health & Wellness Center, University of Colorado, Aurora, Colorado, United States of America; 5 Internal Medicine and Nephrology Department, University Gaston Berger, Saint-Louis, Senegal; Subaltern Health, CANADA

## Abstract

Very few studies have analyzed the influence of the environment, rural or urban, on the notion of good life and subjective well-being in sub-Saharan Africa and none, to our knowledge, has combined qualitative and quantitative methodologies for this purpose. The objectives of this interdisciplinary study were: a) to understand the emic representations of the good life in rural and urban Senegal and; b) to compare the levels and determinants of satisfaction with life between these two populations. This study was carried out in Dakar and in a very isolated rural area in the North East of Senegal: the sylvo-pastoral zone of Ferlo. A total of six focus groups were conducted for the qualitative phase, while the quantitative phase was conducted on representative samples of the populations living in Dakar (N = 1000) and Téssékéré (N = 500). Our results indicate that, against all expectations, life satisfaction is better in the Senegalese Ferlo than in the capital, Dakar. This difference may be the joint result of less meaningful social comparisons and a relationship with nature as a source of stress restoration in rural areas. However, the lifeworld of the rural Fulani of the Ferlo is being undermined by global climatic disturbances, which imposes rapid adaptations of pastoralism; otherwise this activity, that is not only subsistence but also identity-based, may disappear.

## Introduction

Over the last forty years, the issue of well-being has become a major concern for both scientists and policy makers who are gradually adopting this framework to assess and monitor human development beyond simple economic indicators. However, the vast majority of studies conducted within this conceptual framework have focused on WEIRDs (Western, Educated, Industrialized, Rich and Democratic countries; [1]). Few studies have been conducted on the good life, or subjective well-being, in developing countries [2]. In particular, this topic has been very rarely addressed in sub-Saharan Africa [3], especially in rural areas [4].

However, it seems necessary to better understand the relationship of sub-Saharan rural populations to the notion of a good life for at least three reasons. First of all, Africa remains the least urbanized region of the world and the only one in which the rural population still surpasses the urban population [2]. Thus, the implementation of national public policies cannot fail to take into consideration the realities experienced by these populations. Moreover, while the total population of sub-Saharan Africa has tripled since the 1960s, the population living in cities has increased tenfold [5], thus demonstrating the attractiveness of an urban "lifeworld" [6, 7] for rural populations. A comparative analysis of the well-being of these two populations should make it possible to better understand the reasons for the sub-Saharan rural exodus. Finally, as will be detailed below, studies comparing the well-being of urban and rural populations in Western societies have produced contradictory results [e.g. 8]. A shift in perspective, through African populations, may shed light on the controversies in this literature.

Thus, the objectives of this study combining qualitative and quantitative methodologies are to a) understand the emic representations of the good life in rural and urban areas in sub-Saharan Africa, and more specifically in Senegal; and b) compare the levels and determinants of satisfaction with life between these two populations.

### Good life, quality of life, life satisfaction, happiness: Concepts and tools

In the literature, the terms "good life", "quality of life", "well-being" or "happiness" are sometimes used interchangeably [9, 10] despite certain conceptual nuances [e.g. 11]. This lack of linguistic consensus, probably associated with the heuristic fecundity of the concept [12], seems to stem from the common interest shared by researchers working in this field—understanding what it means to live well or poorly in a given environment and at a given time—as well as from the multiplicity of disciplinary perspectives on this object of study and the methodologies used to study it.

Despite some epistemological, conceptual and methodological differences, the vast majority of researchers consider the “good life”, or “quality of life”, to be a multidimensional concept [13–18]. This appears perfectly compatible with the idea, also increasingly widespread, that the best way to measure it would be a subjective assessment of well-being, whether through life satisfaction or happiness [12, 19]. If we therefore consider the good life, or quality of life, as a multidimensional concept that can, and must, be assessed globally through subjective evaluations, the fact remains that no consensus seems to be able to be found as to the very nature of its dimensions. For example, the WHOQOL Group has identified between four and six dimensions: physical health, psychological state, social relationships and the environment [17]—to which can be added the level of independence and spirituality [18]. According to the Wellbeing in Developing Countries ESRC Research Group, in Bangladesh, Ethiopia, and Thailand the factors of a good life are: health, money, children, occupation, family, and home [16]. Finally, the survey called "Voices of the Poor" [10], conducted in 23 countries and giving more than 20,000 people living in poverty the opportunity to express themselves on the notion of a "good life", showed that it is made up of five main dimensions: physical well-being, material well-being, social well-being, security (including peace of mind) and freedom of choice and action.

By crossing the results of these studies, it appears that certain factors of the good life are recurrent: health, material living conditions and social relations. Others seem to be subject to greater variability, according to the lifeworld of populations, i.e. according to their environment, their culture and the concrete implementation of the latter through behavior.

### Urban life, rural life and the determinants of the good life

The link between living in rural or urban areas and well-being is far from clear in the literature. The studies that have analyzed this relationship have mainly used quantitative methodologies—with satisfaction with life or happiness scales—and have mostly been carried out in the most developed countries. Comparing several European countries, Schucksmith et al [20] showed that the differences in life satisfaction and happiness were very small within each country, even if rural life seemed to favor subjective well-being in rich countries while the opposite was observed in poor ones. Sorensen [21], who worked on these same European countries, showed that rural individuals had better subjective well-being scores than their urban counterparts "at equivalent economic level", regardless of the economic level of the countries concerned. In the United States [22], like in China [23], the subjective well-being of rural people seems to surpass that of urban people. In Scotland, a recent study even showed that people living in isolated rural areas had higher levels of life satisfaction than those living in cities or even in non-isolated rural areas [24]. However, in London [25], as well as in the Halifax region of Canada [26], researches showed that people living the closest to city centers had the highest levels of subjective well-being. Of course, the contradictions in this literature raise questions.

Given the complexity of human life, the factors influencing subjective well-being are potentially infinite and cannot be described in an exhaustive list [27]. From its first inscription in the field of psychology, many researchers have shown that characteristics related to the personality of individuals (extroversion, self-esteem, etc.) were very strongly linked to their level of well-being [for a review, see 19]. Similarly, many studies have focused on the impact of socio-demographic characteristics on subjective well-being, highlighting in particular the influence of age—often in the form of a U-shaped curve [e.g. 28]. The examination of the relationship between gender and subjective well-being has resulted in more contrasting results [for a recent review, see 29]. Finally, a high level of education also plays a positive role with respect to the level of well-being of individuals [30], even if it is difficult to clearly distinguish the role of education from its economic impact [31].

Consistent with qualitative surveys on the good life, quantitative studies have shown that material living conditions, health and social relations are major determinants. Material living conditions even seem to be the main determinant of subjective well-being worldwide [30]. This would be particularly the case in poor countries where basic needs are not always met [e.g. 32]. Similarly, the literature concerning the relationship between subjective well-being and health status is consistent: good health is a condition that favors well-being everywhere and at all ages, but particularly in older adults [33]. The literature is also unanimous on the impact of social relationships and social capital on subjective well-being: both directly through family and friendships, and indirectly through the health benefits generated by these relationships [34].

All of these factors are likely to distinguish rural from urban populations and thus explain the differences in subjective well-being observed in the studies. In particular, the economic opportunities associated with urbanity could be associated with higher levels of well-being [35]. However, this advantage of the urban environment over the rural environment has been called into question in developed countries where welfare seems to decrease beyond a certain socio-economic level in large cities [2]: social support could then become an essential component, but it would remain much lower than in rural areas [36].

### The good life in urban and rural Africa

Despite the recurrences—if not "invariants"—observed in the literature on the dimensions of the good life and the determinants of subjective well-being, it is now widely accepted that these vary greatly depending on the culture in which individuals live [32]. For example, being an older adult is not (de)valued in the same way in different societies and therefore does not have the same consequences in terms of subjective well-being [37]. At present, however, the overwhelming majority of work on the good life and subjective well-being has been carried out mainly in North America and Europe [38]. In sub-Saharan Africa, such work is very rare. However, according to WHR [2]: "Sub-Saharan Africa is not only one of the areas in the world with low happiness scores, but also a region in which happiness differences between the city and countryside are most pronounced in favor of city life."

Two types of non-mutually exclusive explanations help us understand why Sub-Saharan Africa is the area of the world with the lowest levels of subjective well-being [39]. First, it is well-known that a country’s level of development has a notable relationship to the subjective well-being of its populations. The most developed societies are best able to satisfy the basic needs of their populations—such as access to drinking water, food and housing [40]. Second, subjective well-being is historically a notion related to individualism [41], understood as an intrinsic value of Western modernity [42]. For this reason, it is not surprising that the most individualist societies—i.e. Western and developed—show higher levels of subjective well-being [19, 43, 44].

Differences in subjective well-being between rural and urban Africa—in favor of the latter—are thought to be primarily a consequence of the higher socio-economic level of urban populations [2]. However, even though material living conditions are a fundamental element of good living and subjective well-being, we saw earlier that each urban or rural environment has its own characteristics and, in the case of isolated rural African environments, cultures that may differ (slightly or strongly) from one region to another. Thus, in Senegal, the sylvo-pastoral zone of Ferlo, which is undergoing desertification and inhabited mainly by Fulani, does not have the same characteristics as, for example, the Casamance rural environment, which is tropical and inhabited mainly by individuals of the Diola ethnic group.

### The present study: The case of Senegal and the objectives

This study is based on work carried out in 2009 by Macia et al [3] in Dakar. This qualitative survey sought to understand what emic factors serve as a basis for "the good life", *Dundu bu baxh* in Wolof, in the Senegalese capital. Through a qualitative study involving focus groups comprised of adults between ages 30 and 35 and between 50 and 55, this study yielded results similar to those found by Narayan et al [10]. More specifically, four major dimensions of the good life that emerged included physical health, social relations, material living conditions and individual psychological characteristics. According to the researchers, all of these dimensions were interconnected in the discourses—via direct, indirect, collateral and contagion mechanisms—and, ultimately affected the individual subjective experience of well-being.

The present study aims to extend this qualitative work to rural Senegal and combine it with a comparative quantitative study between rural and urban areas. It took place in the capital of Senegal, Dakar, and in a particularly isolated rural area in the North-East of Senegal: the commune of Téssékéré, in the sylvo-pastoral zone of Ferlo. In a previous study, Duboz and collaborators [36] showed that the Dakar population is better educated, younger (and thus more often single) than the population of Téssékéré municipality. Furthermore, Dakar inhabitants enjoy greater economic well-being than Téssékéré inhabitants. The young, urban, educated population that lives well in Senegal’s capital is indeed poles apart from the rural, geographically isolated and economically deprived population of Téssékéré.

**Fig 1:**
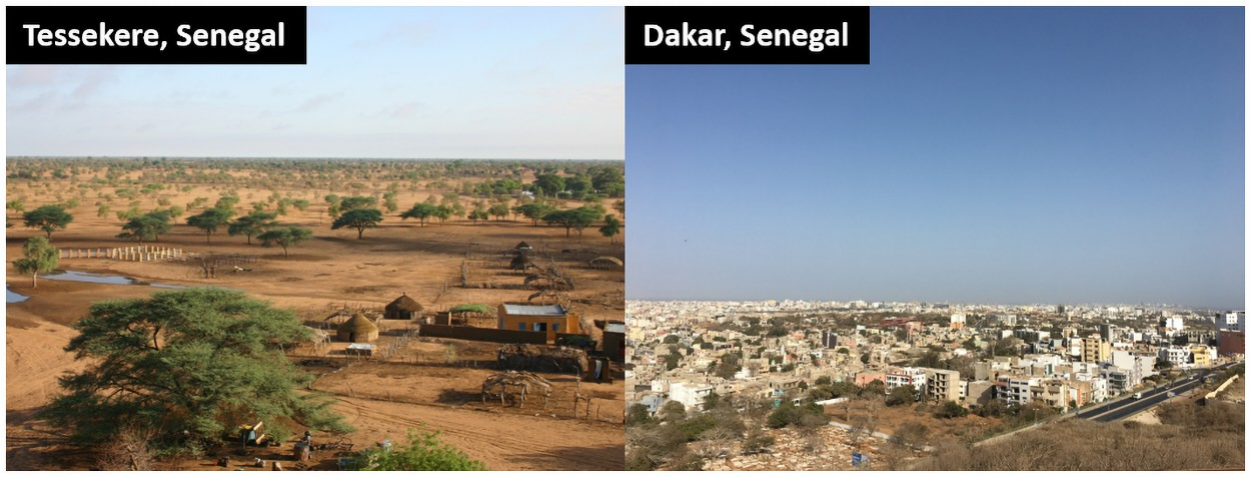
Photographs of the city of Dakar and part of the commune of Tessekere (Senegal) (G Boëtsch; E Macia).

The Dakar environment has largely been described previously [36, 37; see Fig 1 for a visual Dakar-Ferlo comparison]. Let us simply recall that Dakar, the political and economic capital of Senegal, constitutes an archetype of African modernity [45]. The development of the market economy and social classes, the advent of the bureaucratic state or the domination of scientific rationality over symbolic rationalities (to some extent, at least) are all complementary elements in this direction. In the latest ranking carried out by the World Happiness Report [2], Dakar was ranked among the last cities in terms of the international level of happiness (132^nd^/186). On the positive side, it was also the 7^th^ city to have gained the most happiness from its citizens over the last 10 years.

The sylvo-pastoral area of Ferlo, located eight hours drive from the capital (including more than two hours of track) has characteristics very different from that of Dakar. The Fulani, who populate almost exclusively the region, are mainly pastoralists whose life is punctuated by transhumance, necessary to feed the herds during the dry season, which lasts between eight and ten months. In this rural area of the Sahel, living conditions are harsh. There is no running water or electricity. Ecological conditions greatly limit the crops that can be grown, even though "multi-purpose gardens" linked to the passage of the Great Green Wall have been set up in the villages of the region to enable women’s participation in the project, which allow them to benefit from the garden’s production. The great drought of 1973–1974 [*hintande bonde* is the "worst year" in Fulani] was in any case so severe that the crops were definitively abandoned [46]. For health care in the sylvo-pastoral zone of Ferlo, there is a health center in each village (Widou-Thiengoly, but also Amaly and Téssékéré). The first hospital is a two-hour drive away by motorized transport and no one in the area has one except the Great Green Wall forest rangers. This brief description of the commune of Téssékéré and its population illustrates why it can still be described as rural and isolated.

The objective of this work is to compare the levels and determinants of satisfaction with life in urban and rural Senegal, by mobilizing the emic representations of the good life in these two populations.

## Material and methods

This study integrates qualitative and quantitative methods and analyses. This mixed method approach [e.g. 47, 48] appears particularly relevant in the analysis of the good life in Sub-Saharan Africa because it allows both to understand its cultural determinants, which are poorly captured by measurement scales, and to measure the impact of its social and material determinants (age, gender, income, health, etc). The analytic approach was parallel data analysis: data were initially collected and analyzed separately and integrated only at the interpretative stage [49]. As far as possible, equal weight was given to qualitative and quantitative analyses. Qualitative and quantitative results were considered complementary, with each method shedding light on specific aspects of the good life.

For qualitative and quantitative parts of the study, in accordance with the declaration of Helsinki, written informed consent was obtained from all participants. Ethic approval was provided by the Comité National d’Ethique pour la Recherche en Santé (Protocole SEN 13/67).

### Qualitative analysis

The qualitative phase of this study was carried out using the focus group method. This method was chosen because it allows interaction between participants and access to a rich narrative in a relatively short period of time [e.g. 50], while allowing participants not to feel judged by the expert [51]. Furthermore, the good life, as defined here, has never been studied in rural Senegal and focus groups are a very appropriate method for exploring a relatively understudied theme [52]. The fieldwork was carried out in 2019 in the sylvo-pastoral area of Ferlo.

#### Recruitment and sampling

Several questions about the composition of focus groups are still debated in the literature [51]. In the Senegalese context, it seemed more appropriate to form groups that were homogeneous in terms of age, gender and socio-professional categories. Indeed, Senegalese society, like many sub-Saharan societies, is partly organized according to generations [53], with the older adults constituting one of the pillars of social and family organization [54]. Moreover, in Senegal, the roles of men and women are quite distinct, and male domination is obvious. Thus, in order to allow participants to express themselves as freely as possible, we chose to separate young adults (25–35 years) from older adults (45–55 years) and men from women in these focus groups. Moreover, it seemed essential to distinguish between individuals according to their place of residence, since life appears to be very different depending on whether individuals live in the village (i.e., near the borehole, the market, or the health center) or in camps. Thus, eight focus groups were planned in Téssékéré. However, the saturation of discourse was observed very quickly, with the groups always repeating the same thought patterns. Thus, it was decided to stop the fieldwork after six focus groups.

Groups of between 7 and 10 people were thus organized; in total, 50 participants were interviewed. All participants were informed of the voluntary and free nature of their participation, but were compensated 3000 FCFA (4.5 euros) for travel to the interview site. All the focus groups were translated and transcribed in their entirety with the agreement of the participants.

#### Focus groups conduct

During these interviews, special attention was paid to building trust with participants. If it was not possible to recruit participants who did not know each other—at least by name and reputation–, particular attention was paid to the fact that no participant held social position of too much importance (no village or neighborhood chief, no association leader, etc.).

As recommended by Bender and Ewbank [52], a set of questions to guide and direct the discussion had been developed beforehand. Most of the time, only the first question in this guide—"How would you define the good life?—was asked. The interviews were conducted in Fulani and focus groups averaged one and a quarter hours in length, but ranged from 45 minutes to two hours.

#### Data analysis

Data analysis was conducted using the qualitative thematic analysis method described by Mason [55]. The first phase of this analytical method concerns the identification of themes—here, the emic dimensions of the good life. The second phase of this analysis is interpretative and conceptual. The thematic analysis consists of several stages. First, the themes—here, the dimensions of the good life—were identified by reading and rereading the interviews. Both first authors worked independently identifying and naming these dimensions. After discussion between the authors, agreement was reached on the dimensions of the good life. The quotations that best illustrated these dimensions, and their causal relationships, were then selected. Conceptual analysis, on the other hand, is considered more subjective than thematic analysis [56] and consists of an interpretation of discourses. Here, the impact of the physical environment on the good life in rural areas emerged from the readings and rereadings of these interviews and thus constituted the central theme of this part of the analysis.

### Quantitative analysis

#### Population sample for quantitative analyses

This study was conducted from November 2014 to June 2015 on a sample of 1,500 individuals aged 20 and older. The sample was constructed using the quota method (cross-section by age, gender and town of residence) in order to strive for representativeness of the population aged 20 and over living in the department of Dakar and Tessekere. For Dakar, a sample of 1,000 individuals was constructed on the basis of data from the National Agency of Statistics and Demography from the last census (2013). The quota variables used were gender (male / female), age (20–29 / 30–39 /…/ 70 and over with an upper age limit of 100 years) and town of residence. In accordance with the local administrative classification, the towns were grouped by the four *arrondissements* making up the department of Dakar: Plateau-Gorée (5 towns), Grand Dakar (6 towns), Parcelles Assainies (4 towns) and Almadies (4 towns). Practically, this method requires constructing a sample that reflects the proportions observed in the general population. For example, according to the last census, men aged 20–29 living in the town of Medina (arrondissement of Plateau-Gorée) represented two per cent of the population aged 20 and over living in the department of Dakar. The sample was constructed to reflect this proportion and it included 20 men aged 20–29 living in this town. For each town, four doctoral level investigators started out from different points each day to measure and interview individuals in Wolof or French in every third home. Investigators had a given number of individuals to interview (women aged 20–29 / men aged 20–29 / women aged 30–39 / men aged 30–39 etc., in each town) to meet the quotas. Only one person was selected as a respondent in each home.

For Tessekere municipality, a sample of 500 individuals was constructed using the same method and data, but as the area is less geographically extensive, the quota variables were solely gender and age [for more details, see 36].

#### Dependent variable: Satisfaction with life scale

*Satisfaction with life scale* [57] is a popular measure of subjective well-being [58]. It consists of five items, and for each item, respondents select one of seven options (ranging from 1 ‘strongly disagree’ to 7 ‘strongly agree’). Responses are summed up to provide a total life satisfaction score. In the current study, the Cronbach alpha for the sample as whole = 0.725.

#### Health and psychosocial variables

*Self-Rated Health (SRH)*. SRH was measured using a questionnaire with five possible answers: “Overall, would you say that your health is: excellent, very good, good, fair or poor?” For the majority of bivariate analyses and multivariate analyses, this variable was dichotomized. In accordance with Jylhä’s reflection [59] showing a break between good health–“the baseline that does not normally need to have a cause”–and less than good health, the split was made between the answers “excellent,” “very good,” and “good” (scored 0) and the answers “fair” and “poor” (scored 1).

*Stress*. Perceived Stress Scale [60] was used to measure psychosocial stress in individuals. Six out of the ten items of PSS-10 are considered negative (1, 2, 3, 6, 9, 10) and the remaining four as positive (4, 5, 7, 8), representing perceived helplessness and self-efficacy, respectively. Each item was rated on a five-point Likert-type scale (0 = never to 4 = very often). Total scores are calculated after reversing positive items’ scores and then summing up all scores. Total scores for PSS-10 range from 0 to 40. A higher score indicates greater stress.

*Social support*. Social support was measured by asking respondents “If you were in trouble, do you have friends and relatives you can count on to help you whenever you need them, or not?” [61]. Answers were coded 1 when the answer to the question was affirmative, and 0 when negative.

#### Socio-demographic and economic variables

*Economic conditions*. The following question was used as an indicator of economic conditions: “Given your household income, do you feel you … a) live well? b) live okay? c) live okay, but you have to be careful? d) have difficulty making ends meet?” This question, taken directly from Razafindrakoto and Roubaud’s study [62], has demonstrated validity and relevance in eight African capitals, including Dakar, to measure economic conditions in the context of subjective well-being. For the analyses, the answers were coded from 1 (poor) to 4 (prosperous).

*Socio-demographic variables*. Among the socio-demographic data collected during the interviews, four were taken into account for this study: age (20-29/30-39/40-49/50-59/60 and over), gender (male/female) and educational level—defined in accordance with the educational system in Senegal—(0/1-5/6-9/10-12/over 12 years of school).

#### Statistical analyses

Bivariate and multivariate analyses were used to test associations between dependent and independent variables. Bivariate analyses included Chi^2^ tests, student’s t test and ANOVA’s for between-populations mean comparisons. We carried out standard multiple regression analysis to assess the relationship between life satisfaction and place of live by health, psychosocial, socio-economic and demographic variables. All analyses were performed using SPSS software, version 27 (IBM, SPSS software, Armonk, NY: IBM Corp). A p-value < 0.05 was considered statistically significant.

## Results

### The dimensions of the good life

This section is devoted to the analyses of the focus groups conducted in rural area, but few comparative remarks between Dakar and Téssékéré are inserted in order to better understand differences between these environments [for the details of the qualitative study in Dakar, see 3]. Thus, in the Senegalese Ferlo zone, the good life appears undeniably multidimensional, like in Dakar. However, the responses among the groups were much less diverse than what was observed in the Senegalese capital, with all individuals agreeing on two dimensions that are essential for a good life: health and material well-being—or more precisely, good health and the need to be able to provide basic needs for oneself and one’s family. For each group, the most representative definitions of quality of life were presented in Table 1.

**Table 1:** Main definitions of the good life among focus groups.

Group	Participants’ charateristics	Definition of the good life
Group 1	Women, 45–55 y.o. Village	*A good life*, *it is when you have good health and your family is also healthy*, *and you are able to meet the needs for food and other types of care for the family*, *such as prescriptions and schooling for the children*
Group 2	Women, 25–35 y.o. Village	*Health is the basis of a good life*, *but that*’*s not all because I think you have to have the means to provide for yourself*
Group 3	Men, 45–55 y.o. Village	*The first requirement for a good life is good health*. *For a good life also we must have a job because we must have the means to satisfy our needs*
Group 4	Men, 25–35 y.o. Village	*For a good life you need health*, *because without it the person is not physically able to carry out activities such as working to take care of themselves*. *After health*, *I also think that peace and education are needed because they are the means to maintain this good life*.
Group 5	Men, 45–55 y.o. Camp	*A good life is when we are healthy and have the resources to meet our needs*.
Group 6	Men, 25–35 y.o. Camp	*A good life is only what they said*: *to have good health and something to live on*

#### Two main dimensions

Within each group, the same two dimensions of the good life came up repeatedly. First of all, as in Dakar, health appears in the speeches as a necessary condition for a good life for all individuals:

*The basis of a good life is health*, *because when you don*’*t have good health you can*’*t enjoy the rest of your life*

(woman of the village, 48 years old)

*For a good life you need health for yourself and your family members*

(man of a camp, 33 years old)

Being healthy is a necessary condition for being able to enjoy life. Moreover, individual health is not the only one cited in these discourses: it is most often associated with the health of other family members, a condition that is also necessary for a possibly happy existence.

Being in good health also allows access to the second primordial and recurrent dimension for a good life in the Ferlo: material well-being, including of course that of the family as a whole:

*If we are not healthy*, *we cannot think about anything else*, *so it is as if our whole life has come to a standstill*. *In order to work and enjoy life*, *one must first be in good health*

(man of a camp, 50 years old)

*After good health and that of the family*, *you need to have the means to support yourself*

(woman of the village, 45 years old)

In the Ferlo, this material dimension of existence remains largely focused on basic needs, the first of which is food, which occupies a very special place in the discourse of the Fulani of the Ferlo: *"After health*, *you must also have enough to eat"* (man of the village, 52 years old);* "For me a good life is when you have health and enough to eat"* (man of a camp, 29 years old). With regard to food, the main problem highlighted by some groups is that the vast majority of the food consumed in the region—starting with rice, the staple food—is imported, which increases the financial burden associated with this basic need:

*All the basic products we consume come from elsewhere*. *It*’*s hard to have a life where when you wake up with your family*, *the smallest thing you need*, *when you have to go to the corner store [to buy it]*. *When you live like that*, *it*’*s a hard life because you don*’*t control anything in your life*. *We only live on money*, *whereas in the old days with our mothers or grandmothers we rarely saw money because here there was no store and we didn*’*t buy anything*. *Everything we needed to cook was on the spot*

(woman of the village, 48 years old)

While food (and the money to pay for it) is therefore a constant concern for the inhabitants of the Ferlo, this is not the case for housing, which was not mentioned by any individual during the interviews. In the Ferlo, young people build their own hut as teenagers, with the help of their friends, on the plot of their parents (in the village) or in their parents’ camp. This theme fundamentally distinguishes rural from urban people, for whom the question of rent was fundamental to their quality of life.

On the other hand, as in Dakar, it was noted by all rural groups that needs are increasing with what could be called the gradual advent of the market and consumer society. This conversation between younger women in the village is illustrative in this regard:

*Participant n°3*: *There are a lot of changes*, *our current life is very complicated because we have a lot of needs*, *especially us women*. *In the past a woman could have just one good boubou that she wore in ceremonies without any problem*. *But nowadays women go to a lot of parties and they prefer to have a new outfit every time*. *For cooking*, *we only cook rice and other new products that didn*’*t exist before*.

*Participant n°6*: *Yes*, *we have a lot of needs but there is also the fact that nothing is produced here*, *we buy everything*. *When you buy everything*, *you feel like you*’*re spending a lot but we have to because none of the expenses can really go missing*.

*Participant n°3*: *Yes*, *there are many changes that affect even our vital needs*. *We need food*, *clothing*, *equipment*, *health*, *we pay for water*, *we pay for cattle food*.

These "new needs" referred to by these women, but also evoked in the Ferlo men’s groups, concern all spheres of material culture, from dishes (often produced in Asia) to cell phones (and the cost of recharging them), cosmetics or clothing. They are a barrier to a good life in a context where income is limited.

While good health and the satisfaction of needs—above all "basic" needs—are undeniably the two main dimensions of the good life that can be observed in the discourse, social relations were also mentioned by certain groups (two out of six). This was notably verbalized, as in Dakar, through the notion of peace (*Jam* in Fulani as in Wolof). This peace includes both relations with the family and the rest of the community, but also extends to the entire country in an all-encompassing movement. This conversation between younger men living in the village is explicit on this subject:

*Participant n°3*: *After health*, *I think that peace is needed because it is what allows us to maintain this good life [*…*]*.

*Participant n°7*: *For me when you are healthy*, *everything else is negotiable*. *Because if someone claims to have a job or some other activity*, *it is because they are healthy*. *Apart from health*, *I think about peace*. *We tend to neglect this aspect because we are in a stable country but it is precious*. *You have to lose this peace to realize it [*…*]*. *This peace starts in everyday life with the neighbors of the same village and those of the villages next door*.

In spite of this conversation, to claim that social relations constitute a primordial dimension of the good life for the participants would be an over-interpretation, as these speeches represented a minority. This clearly distinguishes rural Ferlo society from Dakar society, where social relations were an invariably verbalized dimension of the good life.

#### Environment, change in the "world of life" and education

The discussions organized in the Ferlo, in absolutely all groups, revolved around a theme that the researchers had not foreseen the sweep in the context of this study: the role of climatic disturbances on the Fulani lifeworld and—therefore—on the good life. This environmental theme was absent from the speeches made by the people of Dakar on the good life.

First of all, all the individuals interviewed in the Ferlo made the general observation of the increasing scarcity of rain and pasture in the region: *"before there were rains but now there is the scarcity of rain in recent years with the lack of grass"* (man from a camp, 53 years old);* "conditions are deteriorating more and more each year*, *the rains are decreasing and pasture is becoming scarcer"* (woman from the village, 27 years old). These environmental changes, perceived as recent and almost always related to the fertile Ferlo "before", greatly affect people’s lives:

*As long as there are problems with water and pasture*, *there can be no good life for the pastoralists we are*. *To have a good life*, *we need water and the improvement of the vegetation to allow us to live in good conditions with our herds*

(man from a camp, 52 years old)

Moreover, these climatic disturbances question the Fulani about the sustainability of their lifeworld. The decrease in rainfall and pasture poses a real identity dilemma: should livestock farming be abandoned when the Fulani identity is based on this activity?

While some women predict the disappearance of animal breeding in the short or medium term (*"I see that animal breeding is going to disappear because conditions are deteriorating more and more each year*, *the rains are decreasing and pastures are becoming scarcer"*, woman from the village, 25 years old), most of the participants emphasize the deep-rooted inclusion of the Fulani culture in pastoralism and the fact that it would be unthinkable to abandon it for that very reason. The following discussion among older men living in the camps illustrates this point:

*Participant n°3*: *The conditions are very difficult at the moment*. *There is not enough rain*, *there is no pasture*, *the dry seasons are getting longer and longer*. *It is all these negative changes that make the life of the pastoralists very difficult*. *In spite of this*, *animal breeding remains our heritage because we have inherited it from our ancestors*.

*Participant n°1*: *The change is the drought which is detrimental to our activity and our way of life*. *I think that we need to change the method because the process has started; and it is getting worse and worse since the great drought that everyone has heard about*. *Since then*, *the livestock industry has not been able to recover from this shock*.

*Participant n°5*: *The solution would certainly not be to abandon livestock farming*, *it*’*s impossible because it*’*s tantamount to denying what we are and what makes us live here*. *The Fulani is made for breeding*, *they do not exist without breeding*.

In the different groups, several solutions—not exclusive of each other—are being considered to improve the lives of the Ferlo pastoralists while maintaining the foundations of the Fulani identity in spite of the desertification of the area. The first of these solutions revolves around the modification of pastoral practices, through the reduction of the size of the herds: *"We must change our practice by reducing the herd because the resources can no longer support the herd"* (woman from the village, 53 years old).

The second solution considered, and discussed within all the groups, is the breeding of new, more productive, breeds of cattle. Here is a conversation with younger women living in the village on this subject:

*Participant n°2*: *Breeding must be changed from the way our grandfathers and fathers used to do it*. *The time when we had to follow the herd all the time*, *without living easily*, *is over*. *It is the education of children that can change things by modernizing the activity so that the pastoralists can benefit from it*. *Alongside this schooling of our children*, *we must also initiate them to animal breeding for the preservation of our culture that we must never give up definitively*.

*Participant n°5*: *We must make a modern breeding where the animals will be less numerous and will be fed and maintained on the spot*. *And since our breeds are not adapted to this type of breeding*, *we must look for more productive ones*, *cows that produce a lot of milk*. *This can allow women to have milk all year round and why not set up dairy farms in the area*.

These transformations in pastoral practices are often grouped together in speeches under the general term "modernization" (in French in speeches). This modernization includes not only the reduction of herd sizes and the breeding of more productive breeds, but also better veterinary monitoring of the animals and the establishment of infrastructure for the packaging of milk and even meat. In order to achieve this modernization of practices, all groups (except for the older men living in the camps) explicitly mentioned the need for children to go to school and be educated. Education must also enable the youngest children to escape from an exclusively pastoral income-generating activity that is now considered insufficient for their well-being. As an older woman living in the village sums it up well:

*We live off livestock farming as our main activity*, *but it is encountering enormous difficulties nowadays*. *I think we have to find something else for our children*. *We need to educate our children so that they can find work elsewhere and succeed to support us*. *We have to change our way of life because it is not possible to continue to live only by breeding*. *Fortunately*, *the current generations are very awake with school*. *Many things will change*.

These speeches show that education appears as the third essential dimension to the good life in the Senegalese Ferlo—or at least as a dimension essential to the well-being of future generations—alongside health and material living conditions. These dimensions are clearly different from the results observed in Dakar, where education was not mentioned as essential to the good life, but where social relations and individual psychological characteristics (including coping strategies) were mentioned by all groups as fundamental dimensions of the good life.

### Satisfaction with life scale in Dakar and Téssékéré

The quantitative results show that average life satisfaction is significantly higher in the rural area of Téssékéré (an average of 23.220 ± 5.743) than in the urban area of Dakar (21.427 ± 5.918; t = -5.585; p < 0.001).

#### Bivariate analyses: Socio-demographic determinants

Bivariate analyses (Table 2) show that women have better life satisfaction than men in both all environments. Moreover, the gender-specific life satisfaction averages were better in Téssékéré than in Dakar.

**Table 2:** Life satisfaction (mean) by demographic, socioeconomic and health related variables (N = 1500).

Variables	Categories	Dakar			Tessekere			Test T	p
		N	Mean ± SD	Test	N	Mean ± SD	Test		
Sex	Men	494	20.664 ± 5.608	t = -4.059; p < 0.001	241	22.382 ± 6.061	t = -3.177; p = 0.002	-3.795	< 0.001
	Women	506	22.172 ± 6.122		259	24.000 ± 5.326		-4.080	< 0.001
Age bracket	20–29 years	424	21.587 ± 5.617	ANOVA	203	23.626 ± 5.675	ANOVA	-4.238	< 0.001
	30–39 years	269	20.807 ± 6.085		116	23.172 ± 5.407		-3.616	< 0.001
	40–49 years	157	20.643 ± 6.104	F = 5.488; p < 0.001	77	21.805 ± 5.815	F = 1.668; p = 0.156	-1.389	0.166
	50–59 years	87	21.885 ± 5.745		52	23.077 ± 6.416		-1.133	0.259
	≥ 60 years	63	24.317 ± 6.122		52	23.981 ± 5.782		0.301	0.764
Education level	None	209	22.124 ± 5.914	ANOVA	376	23.747 ± 5.628	ANOVA	-3.282	0.001
	1–5 years	359	21.571 ± 6.125		87	21.713 ± 5.597		0.197	0.844
	5–9 years	199	21.186 ± 5.645	F = 1.549; p = 0.186	18	21.500 ± 5.803	F = 3.513; p = 0.008	-0.226	0.822
	9–12 years	92	20.804 ± 5.899		13	20.462 ± 6.899		0.192	0.848
	> 12 years	141	20.773 ± 5.733		6	23.167 ± 7.653		-0.988	0.325
Material Well-Being	Have difficulty making ends meet	109	18.009 ± 5.838	ANOVA	73	18.836 ± 5.817	ANOVA	0.937	0.350
	Live OK but have to be careful	161	20.348 ± 5.627		156	23.026 ± 5.652		-4.227	< 0.001
	Live OK	559	21.340 ± 5.524	F = 37.304; p < 0.001	212	23.972 ± 5.165	F = 24.803; p < 0.001	-6.011	< 0.001
	Live well	171	24.906 ± 5.786		59	26.458 ± 4.728		-1.856	0.065
Self-rated Health	Excellent	48	24.292 ± 6.792	ANOVA	24	26.708 ± 6.090	ANOVA	-1.471	0.146
	Very good	100	23.750 ± 5.686		38	25.395 ± 5.011		-1.566	0.120
	Good	549	21.539 ± 5.426	F = 13.494; p < 0.001	223	22.919 ± 5.646	F = 4.180; p = 0.002	-3.166	0.002
	Bad	258	20.380 ± 6.035		175	22.749 ± 5.678		-4.105	< 0.001
	Very bad	45	17.844 ± 7.286		40	22.800 ± 6.111		-3.374	0.001
Stress	Mean	1000	-0.376	p < 0.001	500	-.271	p < 0,001		
Social support	None	163	18.883 ± 5.573	t = -6.105; p < 0.001	40	20.650 ± 5.877	t = -2.973; p = 0.003	-1.777	0.077
	Yes	837	21.922 ± 5.860		460	23.443 ± 5.684		-4.520	< 0.001
Total		1000			500				

While age is related to life satisfaction in Dakar—the over-60s are more satisfied with their lives than the others—the relationship between age and life satisfaction is not significant in rural areas. On the other hand, if we compare the average life satisfaction scores between the two environments according to age group, it appears that those under 40 who are living in rural areas have a higher average life satisfaction score than their Dakarite counterparts. This difference is not significant for those over 40 years-old.

Finally, the level of education is related to life satisfaction in Téssékéré: those with no education have a better life satisfaction score than the others. This relationship is not observed in Dakar.

#### Bivariate analyses: The other determinants of life satisfaction

**Fig 2:**
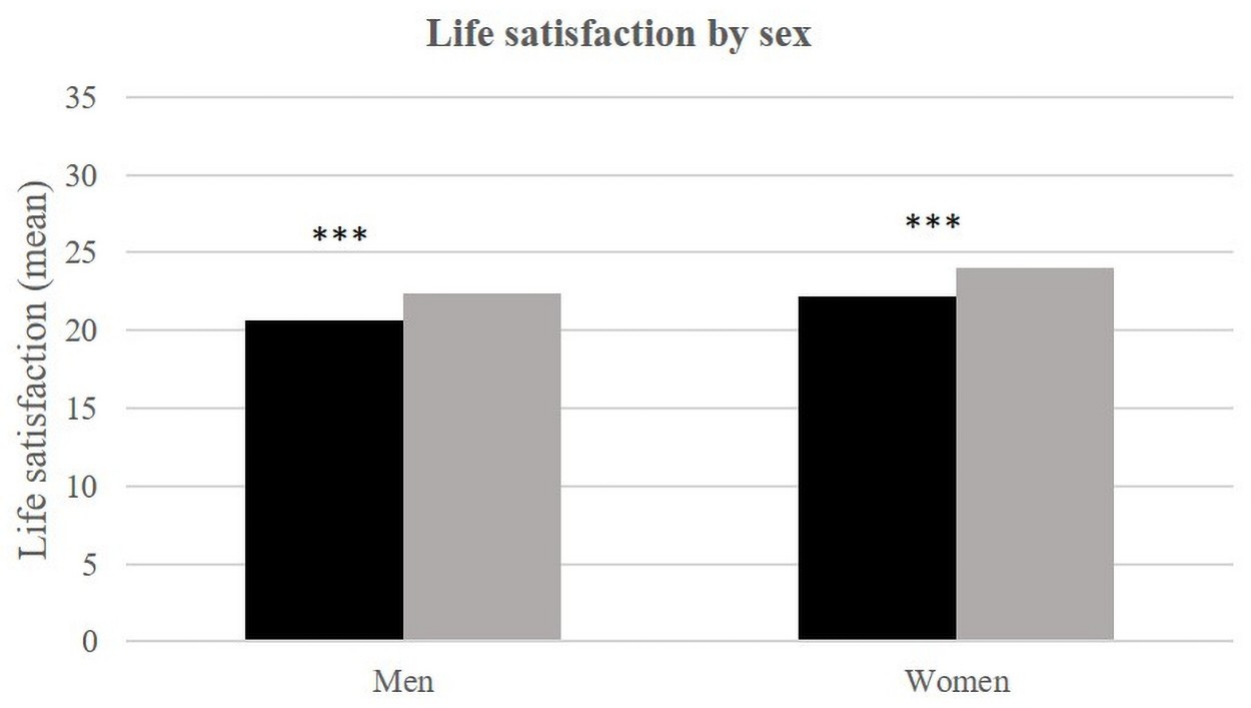
Life satisfaction (mean) by sex and place of life.

**Fig 3:**
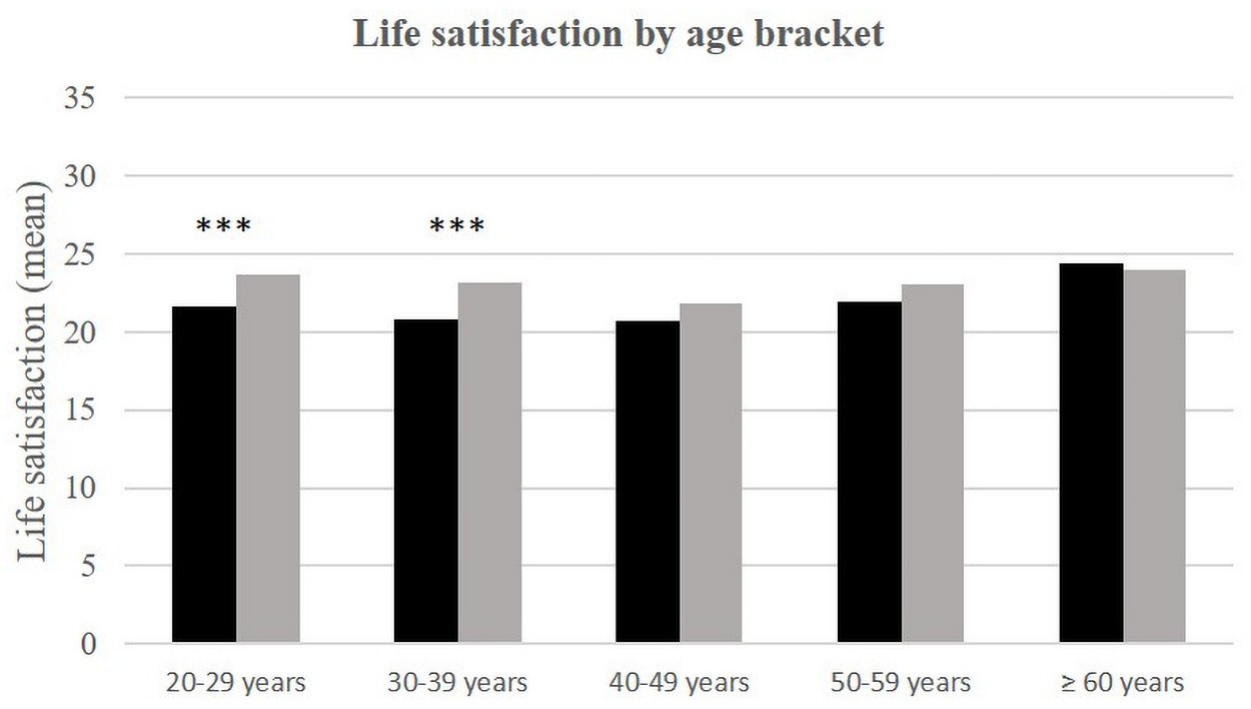
Life satisfaction (mean) by age and place of life.

**Fig 4:**
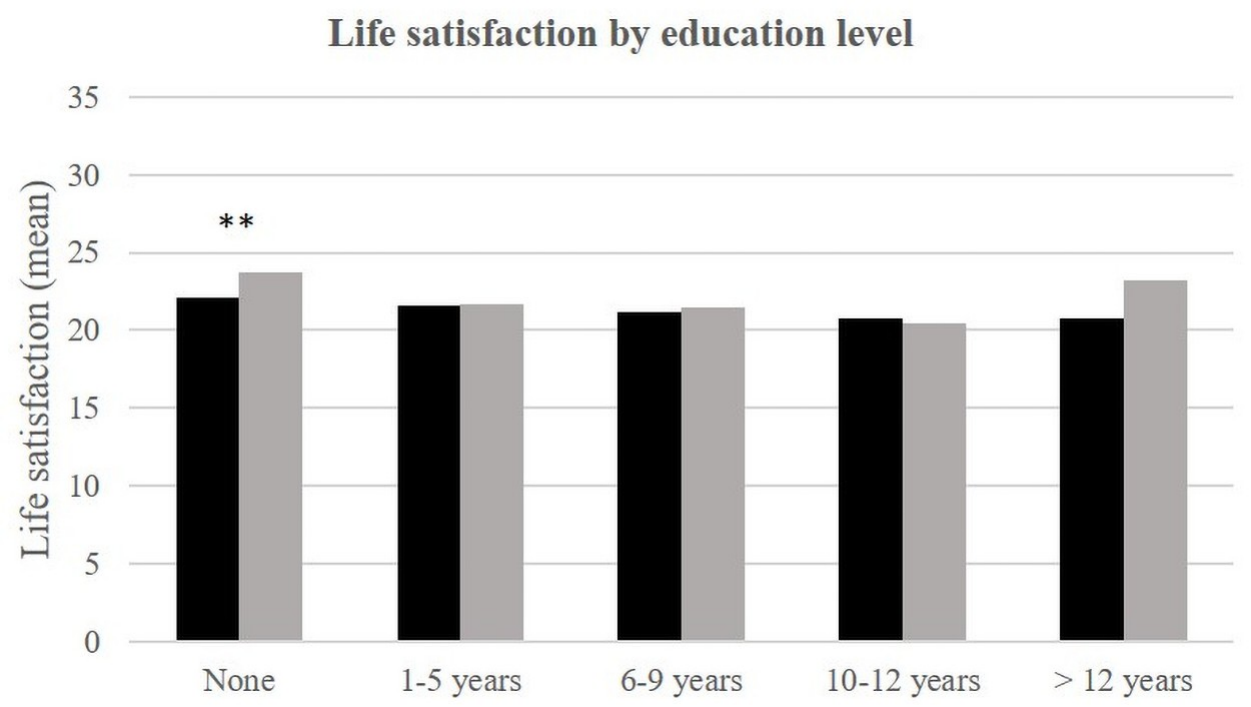
Life satisfaction (mean) by education level and place of life.

**Fig 5:**
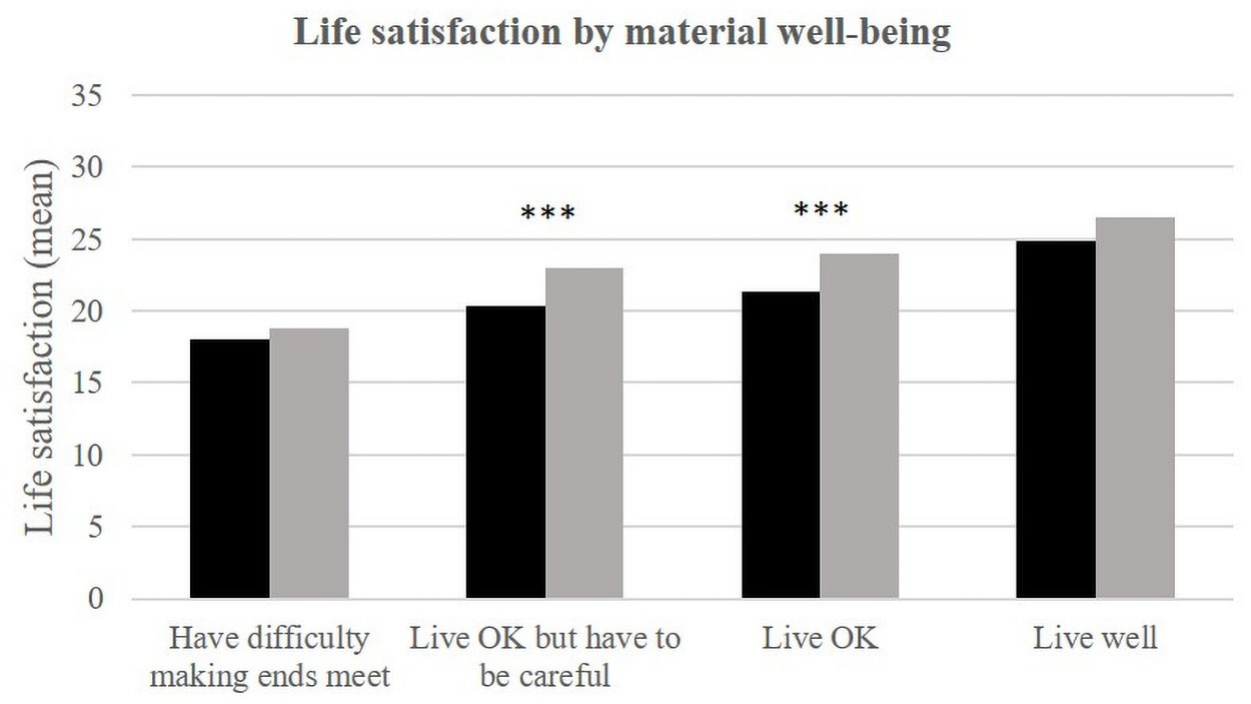
Life satisfaction (mean) by material well-being and place of life.

**Fig 6:**
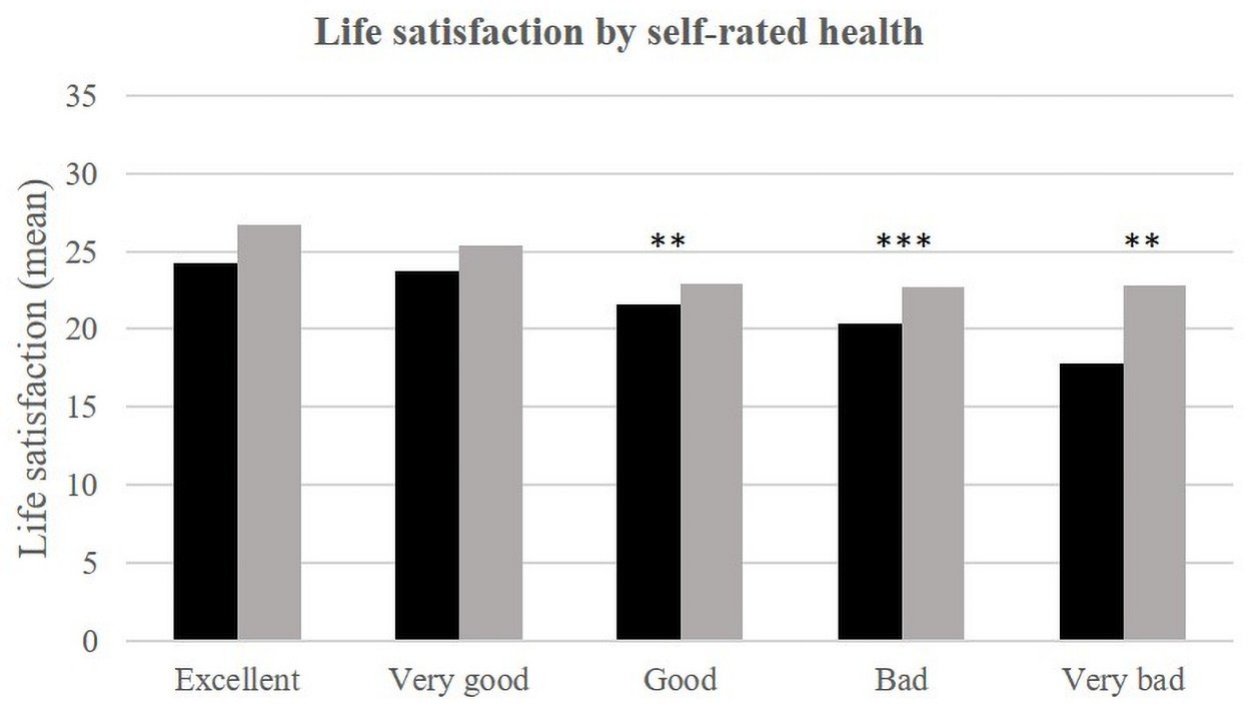
Life satisfaction (mean) by self-rated health and place of life.

**Fig 7:**
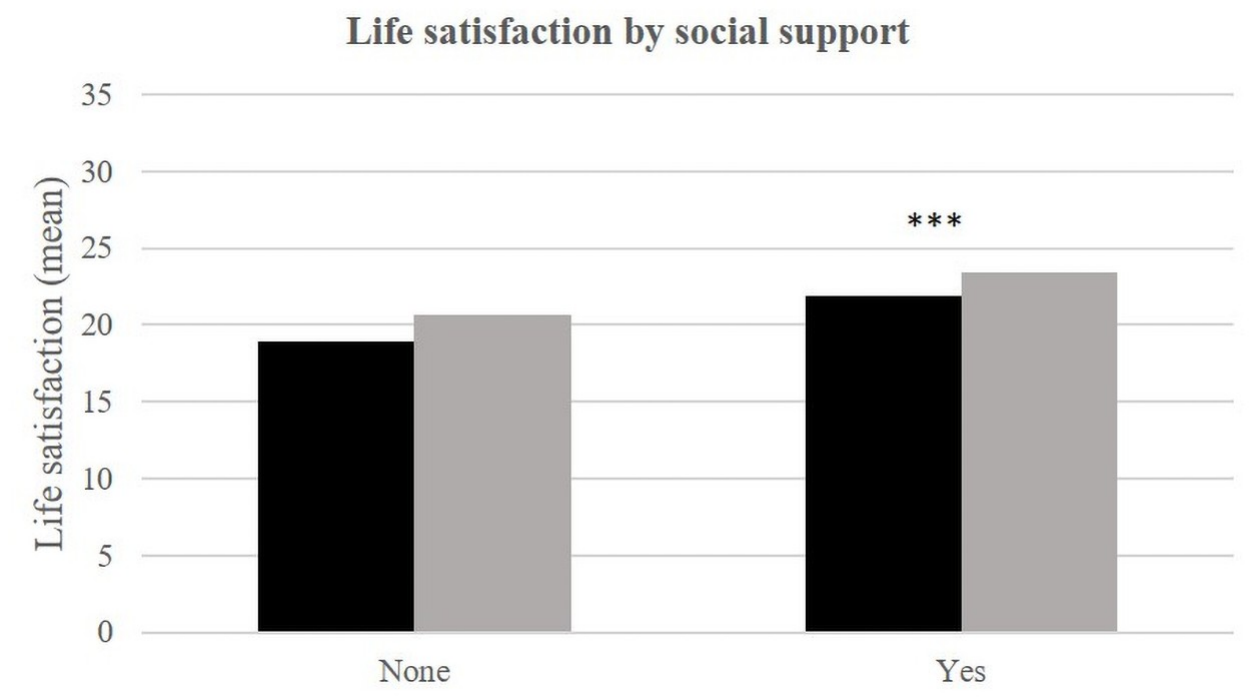
Life satisfaction (mean) by social support and place of life.

**Fig 8:**
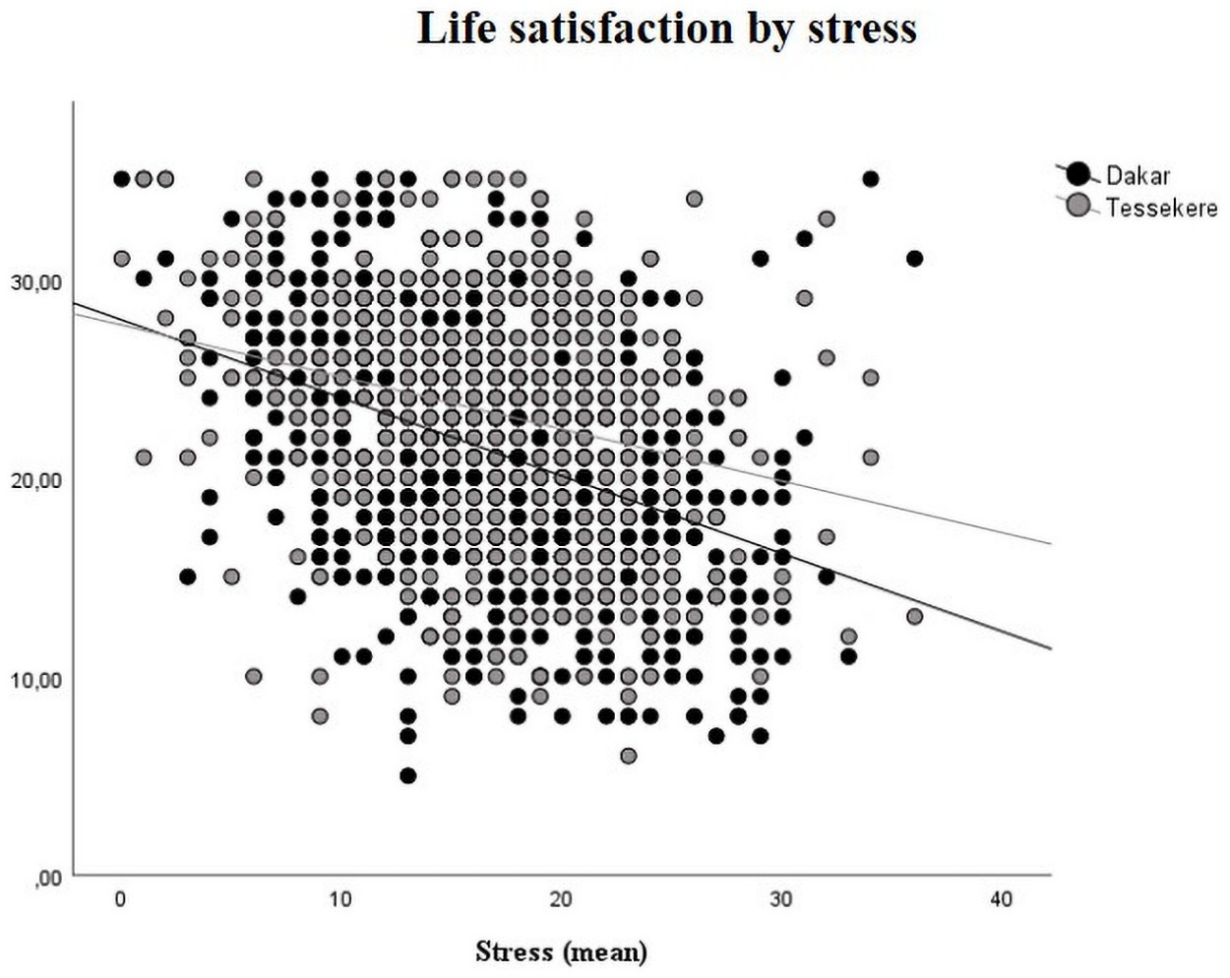
Life satisfaction (mean) by stress and place of life. (N = 1500); * p < 0.05 / ** p < 0.01 / *** p < 0.001.

In each environment, self-rated health, material well-being, social support and stress appear to be significantly related to life satisfaction (Table 2, Figs 2–8).

With regard to self-rated health, only people in good, poor and bad health have lower life satisfaction in Dakar than in Téssékéré. People who consider themselves to be in excellent or very good health have life satisfaction scores that are comparable between the two environments.

People reporting average material well-being ("I’m fine" and "I’m fine but I have to be careful") have better life satisfaction in Téssékéré than in Dakar. No significant difference is observed between the two environments for the extreme categories ("good" and "difficult").

Also comparing Dakar and Téssékéré, the results show that people with satisfactory social support have a higher average life satisfaction score in rural areas. Individuals reporting a lack of social support do not have different average life satisfaction scores between Dakar and Téssékéré.

Finally, stress is associated with life satisfaction in both environments, but to a lesser extent in Téssékéré: life satisfaction decreases less quickly when stress increases in Téssékéré than in Dakar.

#### Multivariate analysis

A generalized linear regression model was applied in order to control the relationship between life satisfaction and place of living by all the variables collected from individuals. The results of this regression (Table 3) show that women, people over 60 years of age, those with an education level of less than 6 years, those who do not report living with difficulty according to household income, those who benefit from social support, those with little stress and those who do not report being in very poor health have better life satisfaction than others. Finally, all other things being equal, this analysis shows that the inhabitants of the commune of Téssékéré have better life satisfaction than the inhabitants of Dakar.

**Table 3:** Regression of life satisfaction on demographic, socioeconomic and health variables (N = 1500).

Variables	Categories	B	CI 95%		p
			Inf	Sup	
Sex (Women)	Men	-1.723	-2.252	-1.194	< 0.001***
Age bracket (≥ 60 years)	20–29 years	-2.195	-3.275	-1.115	< 0.001***
	30–39 years	-2.432	-3.535	-1.328	< 0.001***
	40–49 years	-2.297	-3.456	-1.138	< 0.001***
	50–59 years	-1.341	-2.616	-0.065	0.039*
Education level (> 12 years)	0 year	2.328	1.273	3.383	< 0.001***
	1–5 years	1.208	0.226	2.191	0.016*
	6–9 years	0.815	-0.270	1.899	0.141
	10–12 years	0.226	-1.062	1.514	0.731
Self-rated health (Very bad)	Excellent	3.365	1.683	5.048	< 0.001***
	Very good	2.668	1.220	4.116	< 0.001***
	Good	1.156	-0.037	2.349	0.058
	Bad	0.491	-0.707	1.689	0.422
Material well-being (Well)	Having difficulty making ends meet	-5.352	-6.414	-4.290	< 0.001***
	Live OK but have to be careful	-3.381	-4.284	-2.477	< 0.001***
	Live OK	-2.552	-3.321	-1.783	< 0.001***
Social support (Yes)	No	-1.793	-2.567	-1.019	< 0.001***
Stress score		-0,239	-0.287	-0.190	< 0.001***
Place of life (Téssékéré)	Dakar	-1.218	-1.880	-0.557	< 0.001***

* p < 0.05

** p < 0.01

*** p < 0.001

## Discussion

The quantitative results obtained in this study show that the people of Dakar and the inhabitants of the commune of Tessekere are slightly satisfied with their lives according to the classification of Diener et al. [57]. Although international comparisons are not always easy—most studies are based on specialized population samples (mostly students)—our population samples seem to be above the average life satisfaction levels found in South Africa [63] or Chile [64], but below the values found in Spain [65], Germany [66] or Norway [67].

Overall, the qualitative and quantitative results showed significant differences between the assessments of the good life in urban and rural Senegal. Firstly, the dimensions expressed by individuals during the focus groups were not exactly the same. In Dakar, the classic dimensions of the literature were found: health, material living conditions, social relations and individual psychological characteristics (including coping strategies; [3]). In rural areas, social relationships were not verbalized by the participants, nor were the psychological characteristics of each individual. On the other hand, emphasis was placed on the transformations of the physical environment—desertification—and the need for education to transform animal breeding practices and diversify sources of income.

These results must be placed in their environmental and social context in order to be grasped their complexity. Indeed, Dakar can be considered an archetype of African modernity [45] and the dimensions highlighted there correspond to those found in the literature. Between Dakar and Paris, Dhaka or Beijing, the dimensions of the good life are more or less the same—with their particularities, of course—because existence is built around relatively shared centers of interest. A classic pattern would be that one needs to be in good health to be able to work and thus satisfy one’s own needs and desires, as well as those of the family, which facilitates harmony within the household; all of which makes it possible to live a "good life". Among the Fulani of the Senegalese Ferlo, this classic pattern also works. Although the Ferlo Fulani did not directly refer to social relations like the Dakar people did, it may be that these social relations were so obvious to rural people that they did not even need to point them out: barely 8 percent of the Ferlo Fulani said they could not count on a relative or friend in case of need. This hypothesis is further confirmed by the fact that social support appears to be linked to life satisfaction in Dakar, as in Téssékéré. It may therefore be that, in parallel to what Moller and Saris [68] suggest, the effect of social contacts on life satisfaction may be felt more strongly in Dakar, where income levels are higher and concerns about material well-being are less prevalent.

Above all, the classic scheme of a good life among the Fulani revolves around a specific activity that orders the whole society: pastoralism. There is no need to insist on the fundamental relationship between the Fulani and livestock farming, as entire works are devoted to it [e.g. 69, 70]. However, it is necessary to note that, according to the individuals themselves, pastoralism is being undermined in the region. Of course, the current climatic changes are not unrelated to this: the Sahel is considered a particularly vulnerable area to global changes and is in a desertification phase [e.g. 71, 72]. This decrease in rainfall is logically associated with a decrease in pasture and thus with difficulties in feeding livestock. At the same time, overgrazing, due to increasingly large herds, also weakens the local ecosystem. These concomitant events are at the origin of a central concern for the Fulani: how, under these conditions, can the herds be fed? This is not simply a question of subsistence, but also a question of identity because, as all participants pointed it out, the disappearance of livestock farming would be nothing less than the disappearance of the Fulani themselves. The Ferlo population is currently facing this risk, and the only solution they foresee is through a dimension of the good life that was overlooked in Dakar: education. It is, according to them, through education that breeding can be adapted to these new environmental conditions and that is why they seem to want to invest more in it than before.

These environmental and identity problems, combined with the difficulties encountered by rural people in meeting basic needs, suggested a lower average life satisfaction score in the Ferlo than in Dakar. However, the opposite was observed—significantly and robustly across all the analyses carried out. How can we explain this result, which runs counter to the literature, as well as to what the qualitative analysis suggested?

The first explanation, and perhaps the most obvious, is related to the socio-economic heterogeneity of African capitals, which are among the most inegalitarian cities in the world [73]. Thus, even if the higher socio-economic level of the Dakar population suggested a higher average life satisfaction, it appears that, on the contrary, the strong income disparities characterizing Dakar induce immediate socio-economic comparisons that are detrimental to well-being in urban Senegal [8]. In the Senegalese Ferlo, there is little disparity between the material conditions of existence of the different people and it is a—relatively—homogeneous society. The "richest" are not distinguished from the "poorest" by their material assets. Concerning housing, for example, the better-off have at most a "solid" kitchen (i.e. made of bricks) but the huts are the same for everyone. Similarly, no one has access to running water or electricity. No one has a car, at most a motorcycle. In Dakar, the opposite is true: villas costing several hundred million CFA francs rub shoulders with makeshift barracks; carts get stuck in the same traffic jams as luxury cars; etc. These immediate social comparisons can, of course, affect the subjective well-being of individuals who—even if they think they live rather well considering their income—always want more. In rural areas, we have seen that the needs remain simple: to be healthy, to have enough to eat and to be able to work as a pastor.

Finally, it should be noted that all of the factors most commonly associated with life satisfaction in the literature (demographic, socioeconomic, health and psychological variables) do not fully explain the differences observed between urban and rural Senegal. This means that one, or more likely several, variables not included in this study are likely to explain this difference. In view of the results obtained, a central explanatory hypothesis nevertheless appears. Indeed, within the very broad framework of the work carried out on ecosystem services, many studies have already highlighted the importance of contact with the so-called "natural" environment for well-being: contact with nature could in particular play a role in restoring attention [74] and restoring stress [75–77]. This could, at least in part, explain this rural advantage in Senegal: the stress generated by living conditions could be reduced in the Ferlo by daily contact with a restorative nature. Our result indicating a lower decrease in life satisfaction in rural areas than in urban areas with the increase in stress tends to support this explanatory hypothesis. Even if, according to the qualitative interviews conducted, the environmental conditions of the Ferlo are deteriorating (decrease in rainfall, increase in anthropic pressure) and the Sahelian environment appears to be a constraint, the inhabitants of the Ferlo are looking for ways to adapt to these environmental changes in order to develop a sustainable pastoralism allowing the maintenance of the Fulani identity. Is the attachment to the identity and to the Fulani lifeworld (the *Pulaaku*) coupled with an attachment to the territory? This question remains to be discussed. Similarly, the analysis of the impact of ecosystem services and of the benefits provided by daily contact with nature (restoration of stress, improvement of life satisfaction), mostly carried out in Northern countries and not fully demonstrated for rural populations (for a review, see [78]), remains to be deepened.

This study has several limitations. The first is that the quantitative data were collected in 2015, six years ago. The current relevance of these data can thus be legitimately questioned. However, it seems that lifestyles have changed little in Senegal in five years, whether in urban or rural areas—where there is still no electricity, drinking water, or well equipped health facilities, etc. Despite their limitations (methodological, heuristic), the results of the 2015 and 2020 *Human Development Reports* illustrate this relative stagnation of Senegalese lifeworlds: the country is still at the same rank, i.e. 168 Vs 170; with the same life expectancy and expected years of schooling; only the GNI per capita has increased significantly, but with important social inequalities, as explained above. The second limitation concerns more specifically the qualitative comparisons between our data, collected in the Ferlo, and those collected and published in Dakar in 2010 [3]. We chose not to repeat this data collection because the changes we have observed in Dakar since then seem minor in the context of this study on the good life. Nevertheless, this limitation must be taken into consideration.

Despite these limitations, several implications for Senegalese decision-makers can be drawn from this study. First, and even if it is already well established, measures must be taken to reduce social and economic inequalities, especially in urban areas, and to improve the well-being of the population as a whole—and not only that of a privileged few. Furthermore, it seems necessary to promote the development of green spaces in urban centers in order to encourage contact with restorative nature and thus limit the stress associated with urbanity. Finally, it appears that the economic and social development of rural populations must be considered in light of their specific lifeworlds. In the case of the Fulani of the Ferlo, these development strategies must be based on the identity-based activity that constitutes pastoralism. The Fulani themselves formulate development proposals adapted to their lifeworlds. It is undoubtedly time to consider them seriously.

## Conclusion

This study is, to our knowledge, the first to compare the notion of "good life" in urban and rural areas in West Africa, using both qualitative and quantitative methodologies. If only a few elements of this comprehensive and complex study were to be retained, it would be that a) life satisfaction is better in the rural Senegalese Ferlo than in the urban capital, Dakar; b) this difference may be the joint result of less meaningful social comparisons and of a relationship with nature as a source of stress restoration in rural areas; c) the lifeworld of the rural Fulani of Northern Senegal is undermined by global climatic disturbances, which imposes a rapid adaptation of livestock farming under penalty of the disappearance of an activity that is strictly speaking an identity-based activity.

Our study seems to highlight the fact that the environment, defined here as rural or urban, has an impact on subjective well-being in Senegal, independently of the socio-demographic, economic, social and health factors taken into account in this work. Thus, the environment, both physical and social, in which sub-Saharan populations evolve captures other factors influencing well-being. In the future, it will be necessary to carry out work aimed at identifying more precisely the factors captured by the urban-rural dichotomy, such as issues relating to landscapes, pollution or biodiversity. This approach, carried out in sub-Saharan Africa, seems to be able to shed light on the controversies in the international literature on the impact of the environment on well-being, through the analysis of rural lifeworlds that are particularly far removed from Western realities (both urban and rural), where the vast majority of this work is still being produced.

## Supporting information

S1 Data Quantitative database. (XLSX)

S1 File Focus groups transcriptions. (PDF)
