# Supplementary material for: The good life in rural and urban Senegal: A qualitative and quantitative study
Source: PLoS One. 2021 May 27;16(5):e0252134. doi: 10.1371/journal.pone.0252134 (PMC8158971; doi:10.1371/journal.pone.0252134)
Supplement: S1 File — (PDF) [file pone.0252134.s002.pdf]

Focus Groupe Hommes 45-55 ans Campement

E: Qu'est-ce qu'une bonne vie selon vous?

R1:Une bonne vie, c'est quand on est en bonne santé et que l'on a des ressources pour satisfaire nos besoins. Je veux dire quand tu es bien portant et que tu peux gérer tes propres besoins et ceux de ta famille tu peux dire que tu as une bonne vie.

R2: Une bonne vie c'est de pouvoir satisfaire à ses besoins dans leur ensemble. Par exemple avoir la santé pour se déplacer et faire ses besoins sans aide. Mais aussi subvenir financièrement à ses besoins avec ses propres moyens. Quand tu dépends des autres tu as une vie très difficile.

E:Qu'est-ce que tu entends par le fait d'avoir la capacité de s'occuper ses propres besoins?

R5: La capacité de s'occuper ses propres besoins c'est tout. C'est quand tu es en bonne santé et ta famille l'est aussi et tu possèdes quelques ressources. Si tu veux faire quelque tu le fais sans recourir ou solliciter l'aide de personne. C'est aussi être autonome.

R6: La bonne vie c'est être dans un bon état de santé car c'est le fondement de tout. Après la santé avoir les ressources qui permettent de se prendre en charge. Il arrive que les gens mettent l'accent sur le matériel ou d'autres aspects financiers pour parler de bonne vie mais moi je pense que le plus important c'est la santé. Car si on est pas en bonne santé on ne peut pas avoir l'esprit à autre chose donc c'est comme si toute notre vie était à l'arrêt. Pour travailler et profiter de la vie il faut d'abord être en bonne santé. Quelle que soit ta richesse aussi si tu n'es pas en bonne santé c'est rien du tout. On le voit même très souvent des gens très riche qui quand ils tombent malades ils sont prêts à renoncer à toute leur richesse pour retrouver leur santé. Tu vois ce que nous minimisons comme l'état de santé mais un c'est très important en tant que ère fortune de richesse. Pour parler de notre contexte particulier ici je peux dire que nous n'avons pas une bonne vie car nous tous des bergers nous sommes fatigués et nous avons faim par conséquent nous avons un mauvais état de santé. Le fait de suivre les troupeaux c'est très difficile et c'est dur aussi. Parce que chaque jour, tu parcours des quinzaines de kilomètres pour chercher de l'eau et des pâturages.

R 3: Je suis d'accord avec toi que la santé est très importante voire même prioritaire mais de nos jours si tu ne parviens pas à satisfaire tes besoins aussi tu risques d'avoir une vie difficile. Donc à coté de la santé il faut aussi avoir des ressources et des moyens de subsistance.

E:Vous avez insisté sur le fait d'avoir des ressources est ce que vous pouvez expliquer davantage le rapport que cela peut avoir sur la bonne vie?

R4 : Les ressources sont très importantes car à coté de la bonne santé ou bien ce qui entretient la bonne santé c'est l'esprit tranquille et si ton sommeil est hanté par le fait que tu ne peux pas satisfaire tes besoins tu finiras par tomber malade.

R7: Parce que la vie est dure et pour un responsable de famille c'est une grande charge de pouvoir satisfaire ses besoins et ceux de sa famille. Il n y a pas plus difficile que de n'être dans une situation où tu ne peux pas satisfaire les charges qui t'incombent.

R6: Les ressources sont nécessaires car il y a tout temps des besoins à satisfaire ce qui fait qu'à coté de la santé la vie sera meilleure quand on a suffisamment de ressources et que l'on parvient à mettre notre famille à l'abri du besoin.

E:Les autres je rappelle que tout le monde est concerné c'est une discussion ouverte n'hésitez pas à intervenir.

R1:On parle des ressources parce qu'elles constituent nos principales difficultés actuellement. Nous vivons dans une zone sèche où il n y a pas d'agriculture, de jardins, de champs cultivables comme les champs d'arachides et non plus ceux de mil et il y a rien du tout. En plus nos cheptels deviennent de petit à petit et ne nous permettent pas de satisfaire nos besoins familiaux. De plus si le père de famille a 3 ou 4 enfants il partage son cheptel entre ses enfants. Donc les ressources c'est à dire des moyens de subsistances sont indispensables pour une bonne vie.

R3:Tant qu'il y a des problèmes d'eau et de pâturages il ne peut y avoir une bonne vie pour les pasteurs que nous sommes. Pour avoir une vie agréable, il faut de l'eau et l'amélioration de la végétation pour nous permettre dans de bonnes conditions avec nos troupeaux. Si on est sincère avec nous même on sait qu'il n' y aura de bonne vie que si les populations vivent de leur troupeau qui trouvent l'eau et les pâturages nécessaires à son développement. Ici nous ne cultivons pas, donc nous ne vivons que de l'élevage et il n'y a pas d'autres activités à part le fait de suivre leur cheptel. Comme il n'y a pas d'autres activités pour leur permettent de satisfaire leur besoins les populations vivent actuellement une situation difficile économiquement. En plus nous avons beacoup de frais (charges) on paye tout la nourriture, les ordonnances, le transport, l'eau etc.

E: Est-ce que vous êtes d'accord avec les autres? Qu'est-ce que vous pensez pour ça?

R5: Pour ma part je suis d'accord avec ce qui est dit jusque là. Pour avoir une bonne vie il faut avoir de l'eau et des pâturages en quantité et en qualité pour l'alimentation des troupeaux. En plus de ces ressources pour l'entretien des troupeaux nous avons besoin de plus d'équipement d'école, de dispensaire, des bonnes routes et des projets. C'est en ce moment que l'on pourra parler de bonne vie.

R3: Par rapport à ce qu'il a dit il y a des choses qui commencent à être réglé comme l'eau avec les nouveaux forages. On osait aller nulle part et y rester pendant une semaine car les femmes seules ne pouvaient abreuver les animaux à cause des distances mais aussi de la manière dont on pouvait avoir l'eau. Je me rappelle une année on restait au forage jusqu'à 2h du matin certains même y passer la nuit. Maintenant ce n'est plus un vrai problème car les forages sont nombreux et les distances ne sont si grandes. C'est pourquoi pendant la saison sèche froide je vais travailler dans la vallée du fleuve Sénégal dans des cultures maraichères où je reste jusque la fin de la campagne et c'est mon épouse qui s'occupe de notre bétail.

R6: En ce qui concerne l'état de santé. Ce n'est pas pour nous seulement, il y aussi l'état de santé de ces animaux. Et si tu as un bon état de santé et ton cheptel est malade. Tu ne vas pas rester tranquille donc quand on parle de santé il faut inclure la bonne santé individuelle, familiale et celle du troupeau.

E:Comme à l'instant le problème d'eau commence à disparaitre. Est ce pour les pâturages vous avez constaté des améliorations avec la présence de la GMV?

R7: Par rapport au problème de pâturage, on ne peut pas parler d'amélioration avec la GMV. Car les parcelles sont clôturées et cela permet aux ressources de régénérer au sein des parcelles. Je trouve que c'est bien car la brousse est en train d'être restaurée petit à petit mais ceci ne règle pas le problème des pâturages. Car les pâturages qui proviennent de ces parcelles ne suffisent et on ne peut pas nourrir un troupeau de vache à partir de l'importation des charrettes de pailles. L'autre difficulté avec les parcelles de la GMV c'est qu'elles rétrécissent l'espace de pâturage car en clôturant ces périmètres le troupeau ne plus y avoir accès.

R4: Je voulais aussi parler d'un autre problème de l'élevage en dehors de l'eau et des pâturages il s'agit de la santé animale qui devient de plus en plus inquiétante. Pour la santé animale on utilisait des plantes comme médicaments ce qui se fait de moins en moins car certaines plantes n'existe plus mais aussi le fait que les peuls s'orientent plus vers la médecine vétérinaire. Et

notre principal problème c'est avec ces auxiliaires vétérinaires qui pour certains n'ont reçu aucune formation sérieuse. Ces des gens qui vont sur le marché achètent leurs médicaments et commencent à faire des injections. En réalité ils ne soignent pas ils vendent ils sont juste préoccupés par le vente de leurs médicaments. Ce que je suis en train de dire est assez sérieux car cela peut faire des dégâts non pas seulement sur la santé des animaux mais aussi sur la santé des personnes. Imaginez le soi-disant auxiliaire fait une injection aujourd'hui et que demain le pasteur se rend que la vache devient de plus en plus malade il décide de l'égorger pour le distribuer aux voisins ou bien de vendre la viande à un boucher qui le vendre à la population. Tout celui qui consomme cette viande risque de tomber malade car il mange de la viande infectée car la vache a reçu une injection juste la veille.

E: Les autres qu'est-ce que vous en pensez ?

R1: Par rapport à la grande muraille ces impacts ne peuvent être mesurables aussi tôt. Parce que sont seulement les populations riveraines qui vont en bénéficier. C'est largement insuffisant par rapport à la demande comme pâturage. Parce que la grande muraille a une largeur de 15 km et aussi avec les aménagements. Cette exploitation ne permet pas de faire vivre tout le cheptel. Surtout l'exploitation se fera durant la saison sèche où les gens amènent des charrettes pour amener avec eux les pâturages c'est insuffisant par apport à la demande. Il y a quelqu'un qui a évoqué le manque de formation des assistants vétérinaires qui sont en places. Parce que on les apprend juste à injecter des médicaments et ils n'ont pas la capacité à savoir l'utilisation de ces médicaments. Et ils ne savent pas la durée de vie des médicaments dans la bête. Donc ce qui engendre des problèmes. Il se peut qu'on injecte aujourd'hui et demain on tue la bête. Ce sui peut amener des maladies au niveau de cette population.

E:Toutes les difficultés que vous avez évoquées sont-elles nouvelles? Autrement dit depuis quand avez vous commencé à percevoir ces problèmes?

R4: Le problème de l'eau et des pâturages est un problème ancien je peux dire même qu'il est lié à l'histoire de l'élevage tel que nous le pratiquons. Pour ce qui des auxiliaires vétérinaires, les premiers étaient très bien formés et suivi par le supérieur qui étaient des vétérinaires diplômés. Le problème c'est que ce métier est un devenu un business et c'est une pratique commerciale. Par exemple tu peux voir une personne avec qui tu bois du thé chaque jour et du jour au lendemain il achète des médicaments et commence à sillonner les campements pour faire des injections. Ces médicaments qui sont vendus dans les marchés peuvent avoir des

inconvénients pour la santé animale à cause de l'automédication et le manque de formations des gens qui s'autoproclament auxiliaire.

E: Qui est ce qui veut ajouter quelque chose?

R6: En ce qui concerne les maladies de ces bêtes, ce sont des difficultés. Parce qu'un vétérinaire est une personne capable de consulter les animaux afin de diagnostiquer des maladies. Mais non pas des gens dont tu partageais des grandes places et tu le vois un jour devenir un soignant d'animaux sans subir une formation pire ils n'ont même pas fait des études pour pouvoir lire des notices. Cette personne je le considère comme un simple commerçant qui cherche à vendre ses médicaments et sans se soucier des conséquences.

E:Qu'est-ce qui selon vous a diminué l'abondance des pâturages?

R1: Ce qui a diminué les pâturages c'est simple c'est le manque de pluie. Au temps on nous raconte que la saison des pluies revenait alors que l'herbe de la saison précédente n'est pas encore épuisée. c'est parce qu'il pleuvait beaucoup, la saison des pluies était plus longue donc forcément il y avait des pâturages en quantité et en qualité.

R5: Actuellement on compte le nombre de pluies avec les bouts des doigts car il y en a pas beaucoup. La saison des pluies c'est presque 1 mois ou bien 1 mois 15 jours avec un écart de plus de 10 jours entre les pluies. Ce qui fait que l'on ne peut avoir des pâturages en quantité.

R 7: je ne reviens pas sur le manque de pluies et la durée des saisons des pluies. Il y a un autre problème nous n'avons plus d'espace car la brousse est partout habitée. Donc ici il faut intégrer le facteur espace car il contribue au manque de pâturage car le cheptel devient de plus grand alors l'espace se rétrécit à cause de l'augmentation de la démographie humaine et la naissance des villages et hameaux. Il est rare maintenant de faire 10 km sans tomber sur un village ou un campement.

R3: Comme il a dit seulement au départ il y a des pluies mais actuellement il y a la rareté des pluies durant ces dernières années avec le manque d'herbes. Il y a un intervalle de temps qui est important entre les pluies. Cet écart entre les pluies conduit à la diminution de l'herbe qui avait poussé au départ d'où la mort de cette herbe par le manque d'eau. La question du cheptel aussi est très important car nous tous ici savons nos espaces de pâturages ne peuvent plus contenir notre cheptel qui devient de plus en plus grand. c'est normal et compréhensible parce que le cheptel appartient aux humains c'est tout à fait normal quand la population augmente que le cheptel aussi s'accroit.

E: Ces difficultés dont vous vous parlez sont dues à quoi?

R3: C'est Dieu seul qui sait mais nous avons noté beaucoup de changements et l'avancée du désert. Mais nous savons tous que la pluviométrie a chuté. Une seule pluie des années passées peut être égale à la pluviométrie de toute une saison de nos jours.

R1: Comme il y a une augmentation importante de la démographie humaine qui a entrainé l'augmentation du cheptel. Pour ce qui du cheptel il y a changements dans la gestion de celui-ci. Au temps il y avait qu'un seul cheptel pour la famille qui était sous la responsabilité du chef de famille. Mais de nos jours cela à beaucoup changé car l'entretien du troupeau est très difficile ce qui fait que les chefs de famille préfèrent donner à chaque fils adultes sa part pour qu'il le gère lui même. c'est pourquoi nous avons plusieurs cheptels et que chacun cherche à démultiplier son troupeau ce qui a entrainé l'augmentation des cheptels d'une manière générale. Ces changements dans la gestion du troupeau ont eu des conséquences dans la naissance de nouveaux campements car chacun crée son propre campement pour s'éloigner du campement familial.

E:Vous continuer à pratiquer de l'élevage à l'ancienne quelles sont les bases c'est à dire les fondements d'une bonne vie?

R7: La bonne vie pour nous est celle de notre troupeau. Pour cela il faut des pâturages en quantité et en qualité, une bonne santé pour le troupeau. Sans cela nous ne pouvons pas vivre à l'aise.

R6: Un peulh ne peut pas abandonner l'élevage c'est notre source principale de de revenus. Donc les conditions de notre bonne vie, se rapportent aux bonnes conditions de vie du troupeau. Mais avec les changements dont nous avons évoqués ici il faut des réponses à toutes les difficultés que nous vivons actuellement avec notre troupeau. l'eau est presque réglée avec les nouveaux forages qui ont raccourcit les distances entre les points d'eau. Il reste le problème de l'alimentation et de la santé [du troupeau] qui sont aussi très importantes.

R5: Pour avoir une bonne vie ici, les éleveurs ont besoins des appuis ponctuels par rapport à toutes difficultés qui sont soulevées. Maintenant que nous avons des forages il faut l'aménagement des espaces pour faire des cultures fourragères mettre en place des projets pour nous permettre de travailler et de gagner quelque chose pour satisfaire aux besoins de famille.

R2: Notre bonne vie était basée sur la pratique de l'élevage extensif. Il nous fallait des espaces de pâturages des quantités et des qualités pour nos troupeaux mais ça va changer nous en avons

conscience. Et ça va changer et ça change déjà. Donc il faut que les fondements de notre bonne vie. Car notre vie actuelle est différente de notre vie ancienne c'est pourquoi les fondements aussi changent car nous pouvons pas continuer comme avec ce mode de l'élevage c'est bon d'avoir des logements où on habite. Là où tu auras pour scolariser tes enfants. C'est important de vivre dans de grands villages avoir des voisins pour bénéficier des infrastructures.

R1: Moi je pense qu'habiter ensemble [le village] est une solution au problème de gestion de l'espace. Cela peut être une alternative parce que les peulhs n'ont jamais habité ensemble dans de grands campements. Maintenant pour libérer de l'espace, il faut se regrouper pour libérer de l'espace qui sera réservé aux pâturages. Ce regroupement de ces populations pour constituer de gros villages, ça permet de libérer de l'espace. Parce qu'ils voient la pression foncière venir et donc c'est un obstacle.

R6: Comme nous n'avons pas fait d'études, nous n'avons non plus de qualification et nous n'avons pas d'autres ressources de revenus si ce n'est la pratique de l'élevage. Il n'est pas envisageable de se séparer de notre troupeau.

R3: Ce serait difficile pour nous de vivre sans le troupeau. Car c'est l'élevage qui constitue notre activité principale et c'est le troupeau qui est notre richesse. C'est tout ce que nous savons faire et nous sommes socialisés dans cela depuis notre enfance. Car la relation qui nous lie à notre troupeau va au-delà de cet aspect matériel où le troupeau représente qu'un bien. La preuve est que les peuls qui sont éduqués dans ce système socio-culturel veulent tout temps avoir du troupeau même s'ils vivent en ville. D'autres vont jusqu'à acheter du bétail qu'ils confient aux membres de leur famille.

R1: C'est vrai qu'il y a des gens qui vivent une bonne vie sans être des bergers. Mais nous ne pouvons pas vivre une vie normale sans le troupeau sans mener l'élevage car c'est ce que nous connaissons depuis notre enfance et c'est ce que nous avons hérités de nos parents.

R6: Au delà des explications que vous tous avez donné je pense que la réponse est très simple. Un peul ne peut pas vivre une bonne vie sans le bétail. Ou bien s'il parvient à avoir une bonne sans son troupeau et en dehors de l'élevage je crois qu'il renonce à cette identité peul car ce qui fait un peul c'est son troupeau et la relation qu'il entretient avec celui et la communion qu'il entretient avec son environnement.

E: Nous vous remercions aussi de votre présence. Nous sommes de chercheurs et nous intéressons aux modes de vie des populations peuls surtout à des questions liées aux

mutations de ces modes de vie et des conséquences que ces mutations peuvent avoir sur la santé des populations. Une fois de plus nous vous remercions de votre collaboration.

Focus hommes 45-55 ans Widou Centre

E: Nous allons directement aux questions et chacun peut répondre par rapport à sa connaissance et en son domaine, il n y a pas de mauvaise réponse, c'est vous qui savez c'est pourquoi nous sommes là pour vous demander. Selon vous, Qu'est-ce qu'une bonne vie ?

R1:c'est à chacun sa question ou bien ?

E: non c'est pour tout le monde, c’est une discussion quiconque a une réponse peut intervenir ?

R1: Bon moi, je pense nous ne sommes pas des jeunes nous avons vécu plusieurs époques ici. Ce village existe depuis longtemps et c’est en 1956 que nous avons commencé à avoir de l’eau avec l’implantation du forage. Et notre vie a beaucoup changé entre temps.

E: oui, mais d'après tout cela qu'est-ce-que vous pensez comme étant une bonne vie? La question est adressée à tout le monde.

R1 : D'après moi, la vie d’'avant et celle d’aujourd'hui n’est pas la même. Au temps nous étions plus fatigués mais nous avions une meilleure santé. Nous marchions plusieurs kilomètres à la recherche de l’eau. Avant les éleveurs avaient des champs partout, nous mangions beaucoup de mil ; le riz était très rare. Il n’y avait pas beaucoup de maladie, le diabète, la tension et les autres maladies n'existaient pas. Avant la forêt était beaucoup plus abondante, il y a des herbes qui deviennent de plus en plus rare. Nous étions aussi plus à l'aise par rapport au gouvernement nous ne payons pas de gaz (carburant motopompe forage), ni des ordonnances dans le régime Seghorienne de même que Diouf. Mais à l'arrivée de Abdoulaye Wade ça commençait à changer, y'a peut-être des avantages mais y'a aussi des inconvénients qui nous ont fatigués comme les gaz et les ordonnances et cela est à payer, tu as compris et tout cela n'existait pas.

E: OK, donc qu'est-ce-que vous aussi vous voulez ajouter ?

R3: alors je voulais revenir sur la question si je ne trompe pas tu as demandé c'est quoi une bonne vie, n'est-ce-pas ?

E: oui c'est ça effectivement.

R3: donc il faut que tu nous aides un peu, de le rappeler sinon nous risquons de ne pas répondre véritablement à la question, si quelqu'un fait hors sujet tu essayes de lui réorienter. Un premier critère d'une bonne vie, c'est d'abord la santé, je peux dire qu'avec la santé on a une meilleure vie. Pour une bonne vie aussi il faut avoir un travail car nous devons avoir des moyens qui nous permettent de satisfaire à nos besoins. Moi en tout cas c'est ce que je pense.

E: les autres qu'en pensez-vous?

R5: il est difficile de parler d'une bonne vie car il y a beaucoup de changements que nous sommes en train de vivre et qui ont des conséquences sur la qualité de nos vie par exemple il y a des vieux (personnes âgées) ici qui sont plus saints que nous. Parce que qu'ils ont vécu l'ancienne période où les conditions de vie étaient meilleures. Je pense que si nous voulons parler d'une bonne vie, la santé est primordiale. Deuxièmement, il faut aussi de la connaissance c'est-à-dire apprendre, s'instruire pour savoir comment vivre si tu n'as pas reçu aucun enseignement tu ne sauras pas comment s'organiser ou-bien tu ne sais pas lire ni écrire (français ou Pulaar) cela pose problème tu vie comme un aveugle et tu ne pourras pas conduire ta vie.

E: Selon qu'est-ce qu'il faut pour avoir une bonne vie ici ?

R2 : Moi je vais essayer d'aborder dans le même sens les deux autres. Ce n'est pas facile de parler de la bonne vie. Mais je pense tout, tourne ou se base sur la santé. Après la santé aussi il faut avoir de quoi se nourrir. Je ne parle pas de l'argent ce n'est pas le plus important pour moi. Car si tu as cet argent et que tu n'es pas instruit pour savoir comment est-ce que tu peux gérer cet argent tu finiras par tout perdre. Après la santé, la prioritaire c'est le savoir qui nous permet de bien gérer notre vie et de chercher les moyens pour vivre. Pour moi-même pour faire un jardin et

y trouver des produits pour ta nourriture tu as besoin du savoir et de la connaissance. Ce qui est une bonne vie c'est avoir la santé, être instruit et avoir les moyens subsistances.

E: X et X qu'en pensez-vous? Mais aussi chacun par rapport à son domaine qu'est-ce qu'il peut dire comme étant une bonne vie ?

R7: ce qu'ils ont dit seulement (rire). Je vais juste répéter c'est tout à fait vrai ce qu'ils ont dit, la santé d'abord et avant tout. Après la santé il faut aussi bien veiller sur l'alimentation avant de manger de bien voir ce qu'on mange parce que si quelque chose entre dans un ventre ça serait pas facile de le ressortir, donc quand on mange ou boit surtout ce qu'on mange doit être propre, même l'eau doit être propre. J'entends aussi nos pères dirent qu'il n'y avait pas de bouillons comme le « jumbo » et aussi les maladies d'aujourd'hui. Ils mangeaient plus sain, le sucre aussi la même chose a changé. C'est cette différence que l'on voit aujourd'hui différents plats qu'on ne connaissait pas au temps.

E: donc pour vous tous si je comprends bien, pour avoir une bonne vie, il faut la santé et la manière de gérer cette santé en rapport avec notre alimentation ?

R4: Ici nous vivons de l'élevage comme activité principale mais si on faisait de l'agriculture avec chaque famille un champ cela peut contribuer à ce que nous avons une vie meilleure. Quand tu manges ce que tu cultives tu auras une très bonne santé. C'est seulement l'agriculture et l'élevage qui nous permettent d'avoir une bonne vie.

E: Les autres qu'en pensez-vous?

R1: Il faut un accès à l'eau et à des parcelles pour faire de l'agriculture nous allons consommer les produits et nous aurons des pâturages pour nos troupeaux. Quand nous aurons cela notre vie va s'améliorer. Les gens parlent de changements c'est vrai les espaces de pâturages diminuent de plus en plus ce qui rend difficile nos conditions de vie. Il faut savoir aussi qu'auparavant les aliments que nous mangions n'étaient pas mélangés avec des produits chimiques, c'est pourquoi nous avons plus de santé qu'aujourd'hui. Tout ce que nous mangions était cultivé ici le « niébé » était cultivé, les grains de pastèque étaient cultivés sans produit chimique. Il n'y avait pas

d'école, nos ancêtres ne connaissaient pas tous les problèmes que nous vivons actuellement. Certes nous voulons des écoles, pour apprendre le Pulaar et le français et tout genre de connaissance. Nous voulons aussi des machines et des usines où les gens vont travailler pour améliorer leur vie. Toutefois, ces nouveautés s'accompagnent des conséquences sur notre santé car nos aïeuls avaient une santé meilleure que nous.

E: vous avez parlé des changements de l'agriculture, de l'élevage et aussi les parcelles qu'est-ce que vous pouvez dire de plus par rapport aux modes de vie, leurs dynamismes et des impacts sur la qualité de votre vie ?

R4: Par rapport les années passés, nous notons une certaines différence sur notre mode de vie. L'essentiel pour moi, ce qui a plus de sens pour avoir une bonne vie c'est de contrôler ce que nous mangeons et cela on ne peut l'avoir qu'en développant notre agriculture. Je souligne l'agriculture parce que notre principal problème c'est que nous achetons tout ce que nous mangeons ce sont ces nouveaux produits que nous consommons qui sont à l'origine de toutes les nouvelles maladies qui terrassent de nos jours. Par ailleurs même les produits agricoles que nous achetons sont cultivés avec de l'engrais ce qui pose aussi des problèmes de santé.

E: vous avez parlé de l'agriculture, les parcelles et l'élevage mais est-ce que y'a d'autres domaines ?

R3: ce que je voulais ajouter c'est par rapport à l'éducation. L'éducation fait partie des difficultés que nous rencontrons. A part la santé aussi l'éducation est un pilier important pour avoir une vie épanouie. Nous accusons un grand retard sur ce plan. Mêmes les projets qui interviennent dans cette zone viennent avec leur travailleurs parce que sur place il n y a pas de gens qui peuvent assurer les fonctions exigées ce qui est un handicap. Car à la fin de leur contrat ces employés retournent chez eux sans que les populations ne bénéficient de leur expertise alors les projets ne perdurent pas.

E: vous voulez parler des problèmes des écoles ou bien d'une formation professionnelle ?

R3: Je parle des deux. Il nous faut une éducation de qualité pour les enfants et un suivi jusqu'à la formation professionnelle. Ce faisant les jeunes de la localité pourront être recrutés dans les projets ce qui pourra soulager les familles d'un côté. De l'autre côté, ils serviront leur village et resteront ici.

E: Oui X, tu veux dire quelque chose ?

R4: j'ajoute que l'on ne peut pas avoir une bonne vie si nous n'avons pas un travail qui nous permet de vivre. Il faut trouver du travail pour parler d'une bonne vie. Au temps tout le monde s'activait autour du troupeau et on s'en sortait ce qui n'est plus possible maintenant ce qui a des conséquences sur la qualité de notre vie. Pour ne pas être long l'essentiel tourne autour de la santé et du travail pour prétendre parler d'une bonne vie.

R5: les enfants font presque 10 km par jour pour aller à l'école. Si nous n'avons pas ici des écoles cela va continuer à devenir un problème car pour faire suivre une formation qui donne du travail il faut d'abord avoir été à l'école. Ce qui constitue toujours un problème ici.

E: pouvez-vous revenir en détails sur les problèmes liés à l'agriculture ?

R1 Nous n'avons assez de terre cultivable car vous l'avez certainement remarqué il y a un cheptel très important et nous sommes dans une zone d'élevage donc le problème d'espace se pose. Nous avons aussi besoin de l'eau, de plus en plus de forages, des robinets et même de puits pour faire du maraîchage en dehors de l'hivernage. L'hivernage devient de plus court en termes de période et l'eau est un véritable problème.

E: vous avez insisté beaucoup sur l'agriculture, pourquoi en hivernage vous ne fait pas des petits champs dans vos maison?

R1: Nous ne faisons pas d'agriculture pendant l'hivernage pour plusieurs raisons. La pluviométrie est très faible. Les espaces de pâturages se rétrécissent ce qui fait que les troupeaux pénètrent dans les champs.

E: vous parler des troupeaux pourquoi ici il y a plus de bétails ?

R 5: il y a beaucoup de bétails car c'est une zone habitée essentiellement par les peuls dont l'activité principale est l'élevage. De plus les problèmes de pâturages ailleurs font que les gens du Walo viennent s'installer ici. Ce qui fait qu'il y a un surpeuplement humain et du bétail. Le mode de gestion du troupeau aussi est en train de changer. Il ne se gère plus au niveau de la famille au sens large mais au niveau individuel.

Emm: chacun gère ses troupeaux ?

R 1: oui

E: n'est-ce-pas avant chacun avait ses troupeaux à part?

R 8 : Chacun avait ses troupeaux mais la gestion se faisait au niveau de la famille. On voyait même des troupeaux qui se géraient par campement (wuro). Par exemple si nous partageons tous un père et qu'on partage les troupeaux. Chacun essayera d'augmenter davantage sa part.

E: Vous voulez dire que vous avez abandonné l'agriculture à cause de la pression démographique (humaine et animale) ?

R1 : oui, parce que il y a beaucoup de villages. Tu ne peux pas parcourir cinq à dix kilomètres sans tomber sur villages. Certes les peuls ont de tout de temps étaient éparpillés dans leur mode d'habitat ; mais c'est pire aujourd'hui car tu peux voir une famille qui pouvait habiter ensemble mais dont les membres séparent en cinq ou plus petits de campement.

R4: ici dans le *Jeeri*, nous faisions de l'agriculture pendant la saison des pluies. La forêt avait beaucoup d'arbres, le sol était plus fertile et la pluie plus abondante et nous pouvions faire de l'élevage parallèlement sans problème. Actuellement beaucoup de changements se sont opérés la pluie est presque inexistante, les sols sont devenus pauvres et il n'y pas assez d'espace pour faire des champs.

E: pourquoi les gens cherchent à maximiser leurs profits?

R3: il y a le changement dans notre mode de vie. Les chefs de famille ont de plus en plus de charge pour l'entretien de la famille et du troupeau donc si on se suffit du peu on risque de vivre dans l'indigence totale. Je peux dire que nous pas le choix si nous ne maximisons pas les profits on risque de se retrouver dans une situation où nous pourrions même ne pouvoir satisfaire aux besoins primaires de la famille.

R7: Mes prédécesseurs ont évoqués les changements dans le monde social. Avant c'est le chef de famille qui coiffait tout le monde et tout le monde vivaient sous sa tutelle. Mais maintenant, le chef distribue les troupeaux aux nombres de fils équitable. Il donne chacun à sa part et cette part est considérée comme le premier fond de roulement à fructifier.

E : Cela veut dire que dans quelques part il y'a le phénomène d'individualisme, voici des mots clés qui les empêche de se développer et si c'est le cas, pourquoi ? A quoi est dû à cette séparation, pourquoi les gens ne sont plus ensembles ?

R1: je veux dire quelque à ce propos. Aujourd'hui c'est juste que, les visions sont différentes. Les gens ont de plus en plus de besoins qui sont essentiellement réglés qu'avec de l'argent. Chacun cherche à se prendre ses responsabilité en se charge. Les choses se règlent au niveau individuel pour les adultes.

E: je voudrais que l'on revienne sur cette séparation ou bien de cette individualisation.

R3: ce n'est pas de la séparation en tant que telle. C'est juste une stratégie liée aux difficultés que nous vivons. Par exemple nous avons tous constaté une pratique courante d'un père de famille qui vit avec ses enfants dans un même campement. Très souvent ce n'est pas tous les enfants qui s'occupent du troupeau. Alors le père peut décider de donner à chacun sa part du troupeau pour qu'il s'en occupe individuellement. De ce fait, c'est une manière de les responsabiliser chacun sera obligé de travailler pour s'en sortir. Toutefois nous continuons de partager les mêmes repas pour ceux qui habitent ensemble et s'il te manque quelque chose tu peux solliciter les membres

de la famille. Ce n'est pas de la séparation. C'est une stratégie pour faire travailler les jeunes afin qu'ils sachent se prendre en charge car la vie devient de plus en plus difficile. Excusez-moi!

R4: je vais répondre un peu. Il n'y a pas de séparation. Par exemple avant si tu as six (6) enfants c'est seulement 2 ou 3 qui s'activent dans l'entretien du troupeau, et les autres ne fournissent pas beaucoup d'efforts alors qu'ils ont tous les mêmes droits. Donc pour pousser les gens à travailler le père peut décider de donner à chacun sa part. C'est une stratégie qu'ils ont mis en place pour pousser tout le monde à travailler car la situation devient de plus en plus difficile pour les familles il faut que tout le monde travaille.

E: Si je comprends bien, vous voulez dire qu'il s'agit de l'adaptation pour la gestion des troupeaux. Les autres qu'en pensez-vous ?

R5: Ce qu'il faut comprendre à travers sa réponse c'est que par exemple si nous sommes quatre (4) hommes dans la famille, ce qui est juste c'est que nous tous travaillons pour l'entretien du troupeau en partageant peut être les tâches ou bien en se relayant dans des activités telles que l'accompagnement du troupeau dans la brousse, l'abreuvement ou la collecte de paille etc. Souvent il n y a qu'une seule personne ou deux qui s'occupent du troupeau, les autres ne font presque rien et en retour ils veulent satisfaire leur besoin avec le troupeau. Ce n'est pas normal cela créer des frustrations entre les frères. C'est pourquoi les chefs de famille ont trouvé comme solution de partager le troupeau entre les frères pour que chacun se charge de son cheptel.

R1: Moi je pense que c'est parce qu'aujourd'hui, les gens ont beaucoup de besoins. Et parallèlement l'entretien du troupeau devient de plus pénible donc on ne peut plus continuer à vivre à l'ancienne où tout le monde vivait du troupeau familiale. Quand tu as une femme, d'autres charges te reviennent et pour ne pas fatiguer les autres et d'avoir le courage de travailler, tu gère ton foyer avec ton petit cheptel.

E: est-ce que ces besoins existaient avant?

R1: non non! Il y avait les mêmes besoins mais la manière de les satisfaire a changé. Par exemple depuis toujours les gens mangeaient mais nos ancêtres vivaient du mil, du lait de la

viande qu'ils pouvaient avoir à partir de leurs propres activités. Alors que de nos jours non seulement on achète tous les produits vivriers de plus il y a d'autres besoins liés à l'équipement, à l'habillement mais aussi la complémentation du troupeau qui constituent une charge de plus.

Emm: j'ai une question pour comprendre. Si les besoins augmentent de plus en plus et que les familles s'individualisent, que chacun s'occupe de sa petite famille, il y aura une pression sur les ressources pastorales, comment se manifestera le futur de l'élevage?

R6: Je pense que l'élevage seulement ne pourra pas satisfaire tous nos besoins (...). Sur ce plan tout le monde est d'accord. Si vous voyez la manière dont nous vivons vous savez que l'élevage est en difficulté avec un futur de plus en plus incertain.

R1: C'est vrai que nous vivons de l'élevage dont les troupeaux vivaient des ressources naturelles (eau et pâturages). Là vous constater avec nous qu'il n'y pas de pâturages nous sommes frappés par des sècheresses donc le futur de l'élevage est en danger nous tous ici nous le voyons venir. L'élevage aujourd'hui est menacé de partout manque de pluies, décalage des saisons, augmentation du cheptel et diminution des espaces de pâturages.

E: comment voyez-vous donc la vie des peulhs à l'avenir ?

R1: ça dépend de DIEU (ils parlent tous ensemble). Nous laissons tout entre ses mains car à notre niveau nous ne pouvons rien faire.

R5: Une vie très difficile dont nous vivons déjà les signaux. Courir derrière un troupeau qui ne peut même pas te permettre de satisfaire à tes besoins les plus simples. A la rigueur même c'est le pasteur qui fait autre chose (d'autres activités) pour entretenir le troupeau comme les humains car tous devient payant. Nous ne pouvons plus faire de l'agriculture les pluies deviennent de plus en plus rares. Quand tu as un petit champ, les animaux y pénètrent car les clôtures se font avec des branches d'arbres. Pour les pasteurs d'ici, l'avenir s'annonce de plus en plus difficile.

E: Partant de la situation difficile dont vous faites allusion depuis tout à l’heure. Est-ce que pour une bonne vie pour revenir à notre question de départ vous aspirez à de nouveaux métiers où activités autres que l’élevage ?

R8: Nous ne pouvons pas parler d’autres les métiers. Nous ne parlons que des métiers et des activités que nous connaissons. Nous n’avons pas été à l’école tous ici personne n’a jamais appris un métier si ce n’est poursuivre le troupeau ou faire de l’agriculture.

R4: Nous ne n’avons pas d’aspirations en tout cas moi je n’ai pas d’aspiration en dehors de l’élevage. C’est tout ce que nous savons faire. Certes il y a des difficultés qui sont là, réelles, visibles mais je pense qu’il y a juste des efforts à faire. La solution ce n’est pas de délaissé l’élevage pour autre chose mais d’aider les pasteurs à avoir quelque chose tout en continuant à faire l’élevage. Je pense surtout à mettre en place des parcelles bien clôturées, faire de l’agriculture, du maraichage, de l’élevage moderne.

R4: Tu as vraiment raison de décrire comme ça la situation que nous vivons.

R5: Laisse-moi terminer d'abord. Ce que je veux que vous comprenez c’est que nous vivons une situation particulièrement difficile. Nous ne sommes pas à Dakar et nous avons des opportunités assez réduites qui cantonnent nos possibilités autour de deux activités principales à savoir l’élevage et l’agriculture. Donc on n’aspire pas à d’autres métiers pour une bonne vie il faut sauver ce que nous avons et qui fait notre existence.

E: mais tout à l'heure vous parliez de l'élevage modernisé, c'est quoi en fait?

R3: Pour moi moderniser l’élevage ne veut pas dire l’abandonner où aller faire autre chose. C'est par rapport aux difficultés que nous vivons que nous cherchons des solutions tout en restant dans l’élevage. Par exemple rationaliser les effectifs, avoir des bœufs plus productifs, bien nourrit et bien traité, ce qui sera une rentabilité pour notre vie.

R4: Un peulh ne peut pas se séparer de l'élevage. On peut avoir des métiers parallèles cas même mais l'élevage restera toujours notre socle d'activité. Nous n'ignorons pas les autres activités, mais la forêt est grande. C’est juste qu'il y'a de l'accroissement de la démographique humaine et

animale, l'éclatement familles qui entraine de nouveaux hameaux. Ces facteurs mettent l'élevage en danger en ils entrainent une pression sur les ressources pastorales qui tout de même sont réduites par les effets des changements climatiques. Avoir plus de 100 bœufs alors que tu es fatigué tu ne manges pas à temps ni boire comme il faut, ce n'est pas de la bonne vie. Nous pouvons avoir 25 ou 30 bœufs et les entretenir comme il faut tout en y tirant profit pour satisfaire nos besoins et ceux de nos familles. Si nous avons des cheptels raisonnables à mon avis il sera très facile de gérer les ressources de la brousse et on sera moins fatigué par l'entretien du troupeau et on aura une vie meilleure avec un repos car nous pourrons dormir assez et on aura aussi une bonne santé.

R1: Notre qualité de vie dépend de la qualité de l'élevage. Il faut dire la vérité nous ne pouvons pas laisser l'élevage. Ce qui nous manque dans l'activité ce sont des stratégies qui nous permettent de bien mener notre élevage en vivant de cette de activité. Ce qui nous hante et nous empêche d'avoir la bonne vie dont vous parler ce sont les difficultés que vivent l'élevage. je te donne un exemple quand tu transhume tu marches des kilomètres, tu surveille ton troupeau nuit et jour tu es à la recherche de l'eau, du pâturage etc. Comment est-ce que tu peux prétendre à une bonne vie alors que ton sommeil n'est pas suffisant. Tous ces problèmes c'est par ce que le troupeau vit dans des difficultés et nous aussi c'est tout ce que nous avons c'est normal que courons derrière car nous n'avons pas de salaire, ni de pension tout dépend du troupeau. Ce qu'il faut c'est changer les méthodes de travail. Nos bœufs et nos vaches ne sont pas des races qui peuvent produire à ce point. Ce qui aussi est un handicap pour nous. Les bœufs n'ont pas beaucoup de viande ni du lait. Il y'a des gens qui ont amenés ici des races comme des « laadum » et autres. S'ils vendent ici un mouton « laddum» nous nous vendons 4 moutons « peul peul », pour espérer avoir la même somme. La solution c'est d'essayer de diminuer les effectifs et en l'organisant davantage mais laisser complètement l'élevage c'est impossible pour moi.

E: Selon vous quels seront les conséquences de cette modernisation de l'élevage dont vous parlez sur les pasteurs dans leur mode vie au-delà des aspects économiques ?

R1: Moi je pense à la santé car beaucoup de repos peut toujours avoir des inconvénients sur l'individu. De nos jours il y a des maladies nouvelles qui selon les médecins sont liées à l'oisiveté. Celui qui mange sans faire des efforts physiques risque d'avoir beaucoup de graisse, ce qui amène la «tension».

E: les autres qu'en pensez-vous ?

R4: C'est vrai ce qu'il a dit par rapport à la santé je pense qu'il faut un juste milieu. Courir toujours derrière le troupeau sans y tirer profit c'est une perte. Moderniser l'élevage au point de ne passer son temps qu'à manger, boire et dormir aussi peut être fatal surtout pour les pasteurs dont les organismes ne sont pas habitués au repos. Le diabète et la tension sont là alors que nos ancêtres ne connaissaient c'est types den maladies.

R7: Ne pas faire d'activités physiques on sait tous que ce n'est pas bon pour la santé. Car nous connaissons des personnes parmi nous qui sont atteints de diabète mais c'est parce qu'en réalité qu'ils n'effectuent pas d'efforts physiques et dans nos aspirations à la modernisation aussi nous devons tenir compte des conséquences que cela peut avoir sur notre vie surtout notre santé.

E: Nous vous remercions à tous d'avoir accepté de participer à ces échanges. Nous sommes contents d'avoir pu entendre tous les points de vue qui ont été soulevés par tout le monde.

Focus femmes 45-55 ans Widou Centre

E: Il s'agit d'une discussion comme je vous l'ai expliqué tout de suite. Je pose des questions et vous pouvez prendre la parole pour répondre. Nous allons commencer avec une question très simple. Qu'est ce qu'une bonne vie selon vous?

R 1: **La vie a beaucoup changé ici**. Pour parler de bonne vie c'est par rapport à celle que nous vivions ou à celle de nos ancêtres. Il existe une différence entre notre vie actuelle et celle de nos aïeuls. Leur qualité était meilleure que la notre mais nous avons plus inopportunité et de privilège qu'eux. Nos ancêtres vivaient plus simplement la nature était plus généreuse il pleuvait beaucoup ils avaient de bonnes pâturages, un troupeau de qualité et pratiquaient de l'agriculture. Actuellement les choses ont changés nous avons à l'école, les forages et les dispensaires nous sommes plus modernes. Nos enfants savent lire et écrire. Mais nos ancêtres avaient une meilleure vie car ils avaient une meilleure santé que nous. Ils étaient moins préoccupés que nous avec une alimentation très saine. Nous sommes fatiguées car nous vivons dans une sécheresse. Et il y a beaucoup de maladies.

R2: J'abonde dans le même sens qu'elle. La bonne vie dépend de la santé et de l'alimentation. Quand tu as la santé et manges bien et toute ta famille aussi va bien le reste on peut s'en passer car dans la vie personne n'a vraiment tout comme il le veut. Ce qui fait quand on a les fondamentaux (la santé et ce qu'on mange) le reste on s'en remet à la volonté de Dieu. Nous avons plus de privilèges par rapport à nos ancêtres par exemple pour manger, nos mamans allaient aux champs, pilaient le mil. Tout cela n'existe plus tu peux avoir du bon riz ainsi que d'autres aliments en allant au marché. Auparavant les gens voyageaient difficilement dès fois même à pieds alors qu'aujourd'hui nous avons des véhicules pour le transport et même pour aller à la Mecque il y a beaucoup qui y sont déjà allé il suffit juste d'avoir de l'argent il y a des avions.

R9: La base de la bonne c'est la santé car quand tu n'as pas une bonne santé tu ne peux pas «savourer» le reste de la vie. Après la bonne santé et celle de la famille il faut avoir des moyens pour subvenir à ses propres besoins. Nos ancêtres avaient une meilleure que la nôtre mais nous avons la meilleure alimentation car eux ne vivaient que du mil et du lait. Nous avons plusieurs variétés de produits.

R4: La bonne vie c’est quand tu as une bonne santé et ta famille aussi et que vous parvenez à satisfaire les besoins pour la nourriture et les autres types de prise en charge pour la famille comme par exemple les ordonnances et la scolarisation des enfants. Nous sommes aussi plus éveillés que nos ancêtres qui ne faisaient que poursuivre le troupeau. Nous sommes plus ouverts au monde et plus éveillés. Par contre nous sommes plus fatigués car nous avons plus de besoins.

R3: Pour parler de la situation des femmes nous pouvons dire que nous avons une meilleure vie que nos ancêtres car nous sommes moins fatiguées car il n y a plus de champs et on ne pile pas le mil. Le reste quand même c’est la même chose par rapport aux autres activités ou tâches féminines. Sauf qu’avec l’eau il y a beaucoup d’amélioration grâce aux forages. Nous avons aussi plus de moyens et d’opportunités que nos mamans et nos grandes mères car elles ne vivaient que de la vente du lait.

Q: Selon vous comment faire pour avoir une bonne vie?

R8:Nous vivons de l’élevage comme activité principale mais cet élevage rencontre d’énormes difficultés aujourd’hui. Je pense qu’il faut trouver autre chose surtout pour nos enfants. Il faut instruire nos enfants pour qu’ils puissent de trouver du travail ailleurs et réussir pour nous soutenir. Il faut changer nos modes de vie car il n’est plus possible de continuer à vivre que de l’élevage. Heureusement que les générations actuelles sont très éveillées avec l’école beaucoup de choses vont changer. Pour enfin avoir une meilleure que celle de nos ancêtres consistant à courir derrière le troupeau sans vivre correctement. Ce qui sure c’est que les générations actuelles sont obligées de chercher autre chose en plus de l’élevage. Il n y a plus de pâturages. De plus même avec la transhumance on ne sait plus où aller et quelle direction prendre car la situation est partout la même. Il ne s’agit pas d’abandonner l’élevage, mais d’encourager l’éducation parce que le secteur de l’élevage rencontre des problèmes que nous ne pouvons pas régler.

R7: Nous sommes conscientes que l’élevage rencontre des difficultés et qu’il n’est pas possible de continuer à vivre comme cela. L’élevage ne disparaitra certainement pas de sitôt mais tout le monde ne peut plus continuer à vivre de cela maintenant que c’est saturé il n y a plus de pâturages, les saisons des pluies deviennent de plus en plus en courtes. c’est pourquoi nous les mères de familles nous devons encourager la scolarisation de nos enfants et de nos petits enfants pour qu’ils puissent évoluer dans d’autres secteurs. Autre chose aussi pour une bonne vie il faut continuer à mettre en œuvre des projets dans la zone et d’employer les locaux pour les permettre de gagner de l’argent. Au temps nous n’avions pas de jeunes d’ici qui avaient l’école mais

maintenant nous en avons vraiment certains qui ont été même jusqu'à l'université donc le fait de recruter nos enfants dans des projets qui interviennent ici. Le problème de l'eau est un vieux souvenir sauf pour certains campements qui sont encore un peu loin des forages. Pour une bonne vie il faut d'autres projets en faveur de l'élevage. Pour une bonne vie il faut améliorer nos conditions de vie en nous permettant d'avoir plus de revenus pour subvenir à nos besoins.

R5: Il y a un aspect que je voulais aborder par rapport à notre mode d'habitat qui est très dispersé. Je pense qu'il est temps que l'on se regroupe pour créer de grands villages où il y a tout. Quand on habite ensemble on pourra bénéficier de beaucoup de choses mais aussi le commerce sera plus développé et pourra plusieurs choses comme dans les villes parce qu'il y'aura des habitants qui vont donner une vie à tout. Je pense que le mode d'habitat dispersé est aussi un de nos problèmes.

R3: c'est vrai ce qu'elle a dit le problème des peuls, c'est qu'ils ne cohabitent pas. C'est pourquoi on ne peut pas les aider comme mettre à leur disposition des écoles, des jardins maraichers, des dispensaires et même des usines. C'est révolu le temps de vivre seul avec sa femmes, ses enfants et son troupeau sans école, ni dispensaire. Pour avoir une bonne vie il faut instruire les enfants, changer la gestion du cheptel, moderniser l'élevage car il y a beaucoup de changements et les enfants préfèrent aller à l'école. La dispersion des peuls est une contrainte pour améliorer les conditions. Il y a des peuls qui refusent d'assister ou de répondre présents quand il y a des réunions sur le développement de la zone. Ils pensent que c'est inutile. Pourtant, ces réunions ont beaucoup contribué à l'amélioration des conditions de vie. Grace aux projets nous commençons à bénéficier à beaucoup de choses nous ne sommes plus abandonnés dans la brousse avec nos troupeaux. À chaque instant nous voyons de véhicules de l'administration c'est à nous de faire des efforts. Il faut que les peuls comprennent que le monde change et ils doivent changer sinon ils seront en retard.

Q: Pour continuer toujours avec les difficultés que vous évoquiez. Comment viviez-vous le problème de la recherche de l'eau?

R2: C'était un calvaire la recherche de l'eau. Il n y avait pas beaucoup de forage et saison sèche il fallait chercher l'eau pour les personnes et pour le troupeau. On faisait la queue toute la journée pour trouver de l'eau. C'était essentiellement les femmes qui s'occupaient de cette recherche d'eau en plus des autres tâches qui nous attendaient à la maison. C'est pourquoi nous sommes très soulagées maintenant que l'eau devient de plus en plus accessible bien qu'il y a encore des efforts à faire surtout en saison sèche où la demande est de plus en plus grande. Mais

aussi il y a des besoins nouveaux surtout pour nous les femmes de mettre en œuvre des périmètres maraichers. La facilité de l'accès à l'eau potable a rendu les choses de plus en plus faciles c'est pourquoi les mares ne sont plus fréquentées surtout pour les humains. Il y a vraiment des acquis imaginez on buvait l'eau des mares, on faisait notre linge et notre vaisselle nous courions des risques sanitaires très graves. Les personnes sont plus modernes et éveillées, maintenant presque aucune femme en tout cas dans cette zone n'utilise l'eau des mares pour la boisson.

R1: L'eau était une très rare ici il n y avait pas beaucoup de forage il fallait parcourir beaucoup de kilomètres on puisait avec les ânes. En saison sèche on pouvait passer la nuit au forage et quand le forage tombe en panne on faisait du chiffonnage pour puiser vous imaginez comment on peut chiffonner pour puiser une quantité pour remplir une chambre à air. Il y avait des conflits sur l'ordre d'arrivée pour accéder à l'eau. Les femmes était très fatiguées c'est pourquoi elles étaient toutes chétives parce qu'elles étaient épuisées par la recherche de l'eau et les autres tâches ménagères. Elles n'avaient même pas le temps de prendre soins d'elles-mêmes.

4

Q: selon vous quels sont les principaux obstacles à la bonne vie?

R6: Pour moi, les peuls ne sont pas solidaires entre eux. Ils s'isolent et deviennent individualistes. Actuellement, les besoins sont devenus beaucoup plus nombreux et les moyens ne suivent et l'élevage qui constituait notre source principale de revenus est confronté à des difficultés.

R9: Oui, je crois que nous devons faire attention à l'augmentation du cheptel, parce que les conditions ne sont plus réunies pour élever un grand cheptel. Autrefois, il y a la suffisance, mais aujourd'hui, chacun cherche à augmenter son cheptel. Le cheptel doit être réduit et l'éducation doit être encouragée.

R7: La vie est devenue plus difficile, parce que les gens n'habitent pas ensemble, ils sont très dispersés. Il faudrait les rassembler et les faire habiter ensemble. c'est cette dispersion qui entraine une concurrence entre les éleveurs et exerce une pression sur les pâturages.

Q: Qu’est ce que vous appelez l’augmentation des besoins et comment cela pose problème?

R3: Autrefois, on voyait des champs de mil pendant la saison des pluies et les récoltes nous permettaient de survivre pendant quelque temps. Aujourd’hui on achète tout car rien n’est produit ici par nous. Tous les produits de bases que nous consommons proviennent d’ailleurs. c’est difficile d’avoir une vie où quand tu te réveilles avec ta famille où la moindre des choses que tu as besoins il faut que tu vas à la boutique du coin. Quand on vit comme ça on vit une vie difficile car tu ne maitrise rien de ta vie. On ne vit que de l’argent alors qu’au temps avec nos mamans ou grande mère tu voyais rarement de l’argent parce que certes ici il n y avait pas de boutique mais on achetait rien. Tout ce dont on avait besoin pour cuisiner était presque sur place.

R4: Pour moi ce n’est pas que les besoins ont augmenté mais aujourd’hui c’est que l’argent est devenu la base de toutes les transactions et ce n’est pas facile de trouver cet argent. Même l’argent n’a plus une grande valeur au temps quand tu avais 5 000 tu pouvais rester beaucoup jours avec cette somme. Mais aujourd’hui avec cette même somme tu ne peux pas acheter tous les condiments des repas des familles pour une semaine.

R1: Certes il y a ce fait que nous vivons de l’argent qui rend la vie difficile. Mais il y a aussi le manque de solidarité c’est à dire il n y a plus d’entre aide entre les populations surtout au sein d’une même famille.

Q: Comment vivez les changements en cours?

R2: On ne fait que supporter ces changements en essayant de s’adapter pour suivre. Nous devons s’adapter parce que l’élevage est dans des difficultés, la solution c’est d’amener nos enfants à l’école pour qu’ils réussissent parce qu’il y a rien à faire pour gagner sa vie. Les peuls doivent changer de pratique en réduisant le cheptel car les ressources ne peuvent plus supporter le cheptel l’espace pastoral se rétrécit et il y a plus de pluie et de pâturages. Chaque parent surtout nous les mamans nous devons nous occuper de l’éducation de nos enfants. Nous devons encourager la scolarisation de nos enfants pour qu’ils changent le mode d’élevage qui ne peut pas continuer comme cela.

R6: Nous vivons ces changements très difficilement surtout pour nous les mères familles. Car nous avons en charge les besoins de la famille, tout est cher et les revenus ne suivent pas. Ces

changements qui font que les jeunes sont déboussolés et exercent toutes les formes d'activités possibles pour s'en sortir. Cette situation nous le vivons avec nos enfants.

R4: Ces changements ne sont pas que sur plan de l'élevage et des activités économiques mais il y a des nouvelles maladies. Là nous sommes tous malades ici, ce sont des maladies qui n'existaient pas avant et pire ces maladies incurables comme le diabète, la tension, le mal des genoux et des jambes. Les femmes que tu vois là personne ne peuvent plus marcher 5 km à cause de ce mal jambe qui on dit que c'est lié à notre alimentation.

R2: On doit changer l'élevage parce que les conditions ne se sont plus réunies. On ne peut pas demander à un peul d'abandonner l'élevage parce que c'est sa culture. Mais il peut qu'à même s'intéresser à d'autres métiers comme l'éducation, l'agriculture. L'élevage doit être modernisé en créant des fermes avec des troupeaux de races plus productives. Les peuls doivent ouvrir des boutiques et faire d'autres métiers comme des épiceries comme les maures.

E:Comment moderniser le secteur de l'élevage?

R6: Il faut diminuer le cheptel pour la moderniser mais on ne peut pas l'abandonner et faciliter le contrôle du bétail. Les peuls n'existent plus maintenant car ils n'aiment pas l'activité. On a changé même si on ne peut pas laisser l'élevage. Il faudrait diminuer les animaux, créer des boutiques pour vendre.

R1: Il faut avoir un nombre facile à gérer pour faciliter le gardiennage, le suivi, l'alimentation et l'abreuvement. Quand le cheptel est diminué le suivi sera plus et on va gagner du temps qui permettra aux enfants d'aller à l'école mais on ne peut pas abandonner l'élevage.

Focus group jeunes hommes 25-35 ans

E: Dans le cadre cette rencontre nous allons discuter sur un certain nombre thèmes qui touchent à votre mode de vie et aux changements qui ont intervenus. c'est une discussion assez libre nous avons besoin de tous vos points de vue. Le principe est très simple on pose une question libre à qui veut de répondre je rappelle que c'est une discussion. Nous souhaitons aussi enregistrer la discussion mais c'est anonyme c'est juste qu'il serait difficile de relever vos réponses à main levée ici. Si vous êtes d'accord on commence?

La première question est la suivante : d'après vous qu'est-ce qu'une bonne vie?

R1: Une bonne vie, pour moi c'est difficile de dire exactement car c'est très variable. Il arrive des moments où a une bonne vie et d'autres périodes où elle peut être très difficile. Quand tu prends par exemple la sécheresse la vie sera très difficile, par contre pendant l'hivernage on assiste à des meilleures conditions de vie car y'aura une bonne alimentation, du lait et de la viande. Et nous sommes plus tranquilles car on se repose de beaucoup de choses.

R4: Pour moi une bonne vie c'est quand on a la santé, la paix et de quoi manger. La bonne vie aussi il y a le repos car quand on a pas ce repos notre organisme finit par tomber malade.

E: Et les autres qu'est-ce-que vous en pensez pour une bonne vie ?chacun peut prendre la parole.

R2: Si votre question c'est par rapport à nos conditions de vie comme vous l'avez précisé au début. Nous avons une bonne vie que pendant la saison des pluies. Car le repos est important dans la vie et on ne peut se reposer que pendant la saison des pluies parce qu'en sècheresse beaucoup de chose devient rare. Tu ne dors pas assez, ni ne mange pas car tu as toujours peur est ce que ton cheptel va suivre, comment est ce que tu dois faire pour s'en sortir on ne peut pas parler d'une bonne vie dans ses conditions.

R3: Pour une bonne vie il faut la santé pour soi et pour les membres de ta famille. Il faut aussi pouvoir satisfaire ses besoins et ceux de ces parents. Après il faut aussi une bonne alimentation et se reposé bien.

R: Nous presque notre problème pour accéder à une meilleure vie c'est le manque d'eau. Nous parcourons entre 7 km à 10 km pour avoir de l'eau. Si nous avions de l'eau c'est ça qui nous aidera à avoir une bonne vie. L'autre chose importante aussi c'est d'avoir un travail qui nous permet de satisfaire nos besoins**.**

E: et toi qu'est-ce-que tu en penses comme une bonne vie c'est comment le nom et tourne en face avec moi.

R3: Une bonne vie c'est seulement ce qu'ils ont dit avoir une bonne santé et de quoi vivre. Le reste je pense que les difficultés de temps en temps sont inhérentes à la vie elle même tout ne peut pas marcher tout temps. Mais quand tu as la santé et de quoi manger, tout le reste c'est un plus.

Em: une bonne vie c'est quoi parle nous, qu'est-ce-que tu travail ?

R3: Moi je suis un éleveur je ne fais que l'élevage. Pour un éleveur comme moi il faut avoir la santé, la force physique pour pouvoir pratiquer ton activité correctement. En plus pour un peul (éleveur) si le troupeau est fatigué on est fatigué avec lui. La bonne vie pour nous elle n'est pas que personnelle, elle implique aussi les conditions de vie du troupeau. Il y a certains qui ont parlé avant moi qui avait dit que la qualité de vie chez nous était variable suivant les saisons je pense qu'il a raison car pendant la saison sèche tout devient difficile ici et nous sommes plus fatigués.

E: qu'est-ce qui renvoie aujourd'hui à une meilleure vie pour un éleveur ?

R7 : Ce qui hante la vie d'un éleveur ce sont les conditions de vie de son troupeau, s'il y a assez de pâturage, d'eau et que le troupeau est en bonne santé nous sommes tranquille comme en saison des pluies on a plus de repos, on mange bien et on dort suffisamment. Par contre si nous n'avons pas cela notre vie devient difficile.

R5: Il nous faut de l'eau suffisamment et pas loin de nos habitations, des pâturages, faire des projets où pouvons avoir des légumes pour une bonne alimentation, nous construire des écoles pour les enfants et des hôpitaux pour se soigner. Quand on a cela je pense que l'on sera tranquille.

R4: Les gens parle de santé, de bonne alimentation on ne peut pas avoir tout cela sans avoir au préalable l'esprit tranquille. Et ici nous sommes des éleveurs à cent pour cent (insistance) si le troupeau vit des situations difficile comme en saison sèche on perd notre tranquillité on se repose pas, on ne mange pas et on se dort pas. Si tu ne fais pas tout tu vis la même chose qu'une personne malade. Donc pour une bonne vie il faut commencer par le commencement car santé ou alimentation ou bien repos tout cela dépend de la situation que nous vivons avec notre troupeau. C'est comme une mère avec son nourrissons elle ne peut rester tranquille sachant que le bébé pleure; elle est obligée de voir s'il est malade, ou il a faim ou bien qu'est ce qui ne va pas. Avec nos animaux aussi c'est presque la même chose quand le troupeau va bien tout est là. Le pasteur est tranquille et pourra se reposer, chercher à manger et s'habiller bien. Cela est visible déjà dans nos villages ici. Pendant la saison sèche tu ne vois personne, certains sont maigres alors que pendant la saison des pluies la vie reprend son dynamisme et les gens sont très bien.

E: il y a certains qui ont évoqué l'éducation surtout de la scolarisation qu'est que vous pouvez dire à ce propos?

R2: L'éducation vraiment cela manque beaucoup, en même temps nous n'avons pas des dispensaires. Ici il y a des enfants qui ont l'âge d'aller à l'école si nous avions des écoles ils pourront y aller cela leur servira à beaucoup de chose. Quand une personne ne fait pas l'école il est comme un aveugle car il n'est pas éveillé par rapport à beaucoup de chose. Moi je travaille dans l'électricité (installation de l'électricité dans les maisons, réparations panneaux solaires) je sais que si j'avais fait des études en français personne ne pourrait me retenir (je serais excellent). Il faut donner la chance à nos enfants d'y aller à l'école cela va les ouvrir l'esprit. Mais quand il n'y a pas d'écoles on est obligé de trouver de l'occupation en les initiant à l'élevage.

R3:La scolarisation est encore un problème ici surtout pour nous qui habitons dans les campements. Car nous n'avons pas d'école. Les écoles se trouvent à des distances un longues ce qui n'encourage pas les parents et les enfants à fréquenter l'école. D'un coté aussi c'est de notre faute car on continu d'habiter d'une manière dispersée et le gouvernement ne peut pas mettre l'école dans chaque maison (rire).

Em : sans le repos qu'est-ce qui vous empêche à vivre une meilleure vie ?qu'est-ce qu'on peut tenir aujourd'hui comme étant des indicateurs qui renvoient à une vraie bonne vie chez les peulh? Et cette question ça concerne tout le monde.

R7: Pour moi il y a aussi l'entre aide entre nous. Il y a de moins en moins de solidarité entre les habitants et même au sein des familles. Ce qui fait qu'ici chacun ne se souci que sa propre situation tu peux voir deux frères l'un a les moyens mais n'aide pas l'autre qui vivote avec sa petite famille. Autrefois nos parents disent qu'il y avait la possibilité de prêter des vaches laitières à des familles qui n'en avaient pour les permettre d'avoir du lait. Les gens s'entraidaient vraiment au sein des familles. Certains qui avaient les moyens soutenaient ceux qui n'en avaient au point qu'il était difficile de voir les gens dans certaines situations car la famille, les amis ou bien les voisins venaient en aide aux nécessiteux. Alors que de nos jours les rares qui aident ils le font d'une manière ostentatoire est un «*dimo*», une personne digne aime la discrétion sinon cela ne vaut pas la peine.

R1: Ce qu'il a dit est vrai mais moi je préfère parler d'autres choses qui va vraiment nous permettre de souffler et d'avoir une bonne vie. D'abord l'eau est quelque chose de vitale pour nous, même si les pluies ne sont pas abondantes et les saisons deviennent irrégulière si nous avons l'eau en permanence et non loin on peut faire beaucoup de chose avec. Au-delà de l'élevage on peut faire du maraichage. Par rapport à une bonne vie il faut aussi des écoles et des dispensaires.

E: vous parliez d'entre aide comment cela se passe ici?

R2: l'entre aide est toujours une bonne chose car aucune personne ne peut se suffire à elle seule quelle que soit sa richesse. Les peuls sont connus pour être une société qui s'entre aide en tout cas selon nos ancêtres mais de nos jours on voit que cela diminue. Je pense que c'est parce que la concurrence est très difficile. Je veux dire les gens non pas beaucoup alors que les besoins sont nombreux c'est pourquoi on calcule tout et quand on calcule on pense que quand on aide on devient pauvre. Les gens ici surtout ceux qui sont riches ils aident peu.

R4: Je pense qu'avec l'entre aide on s'en sortira plus. C'est à dire le vivre ensemble peut être avantageux pour nous. La dispersion de notre mode d'habitat est aussi un problème à l'entre aide. On début la grande famille habitait ensemble et tous les problèmes étaient géré par les

responsables de familles. On constate que les grandes familles se sont disloquées et chacun vit de son coté les gens ne se soucient plus des aux autres. Quand les gens n'habitent pas ensemble on devient insensible aux difficultés des autres et on ne s'entre aide pas. Tous les problèmes que nous sommes en train de poser on pouvait les régler si on habite ensemble et que l'on s'entre aide.

E: Qu'est-ce que tu en pense toi, est ce que quand vous êtes ensemble ça peut améliorer votre vie?

R3: Bien-sûr ça peut améliorer notre situation vraiment. Par exemple on parle de l'État et des projets on ne peut nous aider dans cette situation chacun se lève et crée son propre village. Comment on veut que dans chaque village qu'il y ait tout ce n'est pas possible aucun projet ni aucun gouvernement ne peut le faire. (ETAT) La solution c'est d'habiter ensemble former de grand village ça nous aide à avoir de l'eau de s'entraider et d'avoir accès dans les dispensaires avoir des écoles .tout cela ne peut se faire temps que nous ne sommes pas ensemble. Même pour l'élevage il n y pas plus de brousse à cause de ces villages créer à tout va.

R2: Pour ce qui est de l'entre-aide je te donne un exemple si je pars dans la brousse et que je voie des troupeaux de quelqu'un qui habite ici je les ramène, mais si j'ignore vraiment à qui ces animaux appartiennent là je ne pourrai pas réagir vraiment mais si nous habitions ensemble je pourrai reconnaitre les marques des animaux.

R7: Dans les familles aussi, tu peux voir quelqu'un dont la taille de troupeau est très grand. Il creuse son puit, achète un véhicule pour chercher des pâturages pendant la saison sèche mais il ne se souci même pas des gens qui vivent autour de lui ou bien au sein de sa famille. C'est cette insensibilité que nous vivons aujourd'hui qui nous fait dire qu'il n y a plus d'entre aide. Alors qu'au temps les gens se prêtaient même des sacs de vivres pendant les saisons sèches.

E: vous avez parlez de repos et d'alimentation et quoi d'autres qui peut améliorer votre qualité de vie ?

R4: Cela serait l'eau, l'école et des dispensaires. cela pourrait bien apaiser notre souffrance.

R2: Moi je pense à des projets qui vont employer des jeunes d'ici et qui vont permettre de gagner de l'argent.

E: pourquoi l'école et dispensaire ?

R4: Si nous avons l'école sans l'eau, nous irons à la recherche d'eau et là nous n’allons pas bien étudier, il faut d'abord qu'on règle le problème de l'eau et après on installe les écoles et les dispensaires aussi c’est pour que les populations puissent se soigner sans se déplacer.

E: donc d'après tout ce que vous venez d'évoquer, qu'est-ce qui est votre problème majeur qui vous empêche de vivre dans de bonnes conditions ? Ce qui est différent de ce que vous avez déjà évoqué.

R 7: Souvent les écoles sont loin car il y a d’écoles que dans les bourgs et les enfants des campements font des vas et viens pour se rendre ans dans ces écoles. Il y a quelques écoles dans certains campements mais elles ne sont pas fonctionnelles car il n y a pas de personnel.

E:Comment cela peut être un problème?

R 5: si l'école est loin et on ne peut pas aller chaque fois et revenir on risque d'abandonner et on n'apprend autre chose.

E: les autre ce qu'il vient de souligner vous en relevez d'autre choses ?

R 2: je pense aussi que la transhumance est aussi un problème. Le fait par exemple que nous sommes des nomades peut nous empêche souvent de bénéficier à certains structures. À une période de l’année certains hameaux sont désertés on y trouve personnes.

E: vous tous vous avez parlez de l'école qu'est-ce que ça vous apporte en fait ?

R 2: L'école nous permet d'étudier, d’accéder à la connaissance. Quand on apprend nous allons avoir de la connaissance pour nous même et de nous éveiller par rapport à beaucoup d’autres choses.

R 4: Avec l’école on pourra lire, écrire. Même si on se limite à cela lire et écrire seulement c’est un avantage important pour chaque personne. L’école nous permet aussi de savoir gérer nos propres affaires. Le fait que l’on ait pas fait l’école c’est un grand handicap pour nous. c’est la vie qui demande à pouvoir lire et écrire.

R1: Quand on étudie c'est toujours avantageux. Le savoir est primordial pour tout le monde (...) ça sert à tout moment, ça ne fait que servir. Même si on étudie pas pour trouver un travail ultérieurement. On peut étudie pour sortir de «l'obscurité».

E: Pourquoi vous voulez faire autre chose?

R 6: Parce qu'en plus de l'élevage nous devons nous ouvrir ce qui peut se faire d'autre soit dans les villes soit ici c'est toujours enrichissant de faire autre chose. Cela nous permet aussi de découvrir d'autre manière de faire et de moderniser ce que nous faisions.

R 5: Même dans le domaine de l'élevage, on pourra peut-être ajouter d'autres choses de nouveau. C'était seulement le métier des peul mais aujourd'hui y'a d'autres personnes qui le font. Ils sont moins fatigués que nous et ont beaucoup plus de moyens que nous. Vu que les autres s'investissent dans notre domaine nous aussi on doit s'ouvrir à ce qui se faire ailleurs.

R: Moi j'ai presque 30 ans je voyais les habitants des villages qui se rendaient visite ensemble qui s'entraidaient. Tu allais rendre visite quelqu'un en amenant du lait ou autres choses pour leur faire du plaisir. Mais aujourd'hui ça commence à être rare les gens préfèrent de nos jours de l'argent.

E: donc vous avez parlé tout à l'heure du repos etc. Maintenant comment juger vous la qualité et l'amélioration des activités des jeunes d'ici si vous avez des activités parallèles à l'élevage?

R3: Il n y en a pas, nous ne faisons que l'élevage. c'est pourquoi nous avons besoins des projets qui viennent employés des jeunes d'ici. Nous avons aussi besoins de l'électricité peut être cela nous permettra d'avoir des activités économiques.

R2 : Si tu ne vas pas dans la brousse tu n'as rien d'autre à faire. Ce serait bien aussi d'avoir des jardins maraichers pour cultiver avoir quelque chose à manger et on pourra vendre le reste et gagner de l'argent.

Focus jeunes hommes 25-35 Widou centre

E: Nous sommes là pour recueillir vos points de vue sur beaucoup d'aspects de la vie en milieu pastoral peul. Nous posons des questions car nous voulons apprendre de vous. On est là pour essayer de savoir ce qui ce passe par rapport à votre mode de vie au sens large. Vous êtes détenteurs des connaissances sont les experts de leur propre vie. Nous allons discuter de plusieurs thèmes sous formes de questions qui vous seront adressées à vous tous. C'est une discussion assez libre nous souhaitons que tout le monde y participe en donnant vos points de vue. s'il y a lieu de se contredire aussi n'hésitez pas le faire dans le sens d'enrichir le contenu des discussions. Je demande votre permission pour enregistrer le contenu de nos échanges c'est parce que nous ne pourrons pas tout écrire et on ne voudrait pas aussi que quelque chose nous échappe. Avec votre consentement nous allons enregistrer à la fin nous allons ré-écouter les enregistrements pour les retranscrire. Nous allons procéder à la présentation pour nous familiariser un peu dans le cadre de cette discussion.

E: Nous allons commencer avec la première question. Selon vous qu'est-ce qu'une bonne vie? Tout le monde peut prendre la parole s'il pense quelque chose.

R 6: Avoir une bonne vie, c'est être en bonne santé et pouvoir satisfaire ses besoins et ceux de sa famille. Avoir aussi une bonne alimentation et des activités moins pénibles.

R 4: L'élevage est très dur. Tu passes toute la journée dans la brousse. On ne peut parler d'une vie quand tu passes la journée avec les animaux dans la brousse tu ne manges pas à temps tu bois à peine tu ne discutes avec personne (rires). La bonne vie c'est d'avoir un travail descend qui te permet de satisfaire tes besoins dans la tranquillité. Avoir une boutique par exemple c'est moins fatigant tu gagnes ta vie tout en restant au village parmi les personnes et non dans la brousse avec les animaux pour ne rien gagner du tout.

R 6 : D'après moi, pour une bonne vie il faut d'abord la santé. Après la santé aussi il faut avoir les moyens à satisfaire les besoins, par exemple, si tu es malade tu ne peux avoir une bonne vie. Voilà, avoir un métier pour satisfaire les besoins familiales par exemple et les besoins personnels. Pour moi on ne peut pas envisager une bonne vie sans une santé.

R 3: bon, pour moi une bonne vie il faut la santé qui est le fondement car sans cette santé la personne n'est pas physiquement apte pour mener des activités comme avoir travaillé pour se prendre en charge. Après la santé aussi je pense il faut la paix et l'éducation car c'est elles qui permettent d'entretenir cette bonne vie. Pour une bonne vie aussi il faut vivre en bon termes avec sa famille et ses voisins.

E: Les autres qu'est-ce que vous en pensez?

R 1 : «Celui qui implore à Dieu de le sauver n'est pas préoccupé par le gain». En d'autres termes si nous n'avons pas la santé on ne peut prétendre à autres choses. La première chose pour une bonne vie il faut avoir la santé puis avoir des ressources qui nous permettent de se nourrir, de vêtir et d'avoir un bon habitat.

R 7 : Pour moi quand on est sain tout le reste est négociable. Car si quelqu'un prétend à travail ou à une autre activité quelconque c'est parce qu'il a la santé. Mise à part la santé je pense à la paix nous avons tendance à négliger cette aspect car nous sommes dans un pays stable mais qu'elle est précieuse, il faut la perdre cette paix pour s'en rendre compte. Si tu es sain alors que tu n'a pas la paix tu ne pourras pas sortir pour aller travailler. Cette paix elle commence dans la vie et avec les voisins du même village et ceux des villages à côté. Si tu vis dans une peur ou une menace permanente tu n'es pas libre donc tu es toujours préoccupé tu vis la même chose qu'une personne malade.

E: oui et vous autres qu'est-ce que vous en pensez?

R 10: Moi j'insiste sur les moyens de subsistance vous avez tous avez parlé de la santé. j'insiste sur une forme de maladie pire que celle que nous connaissons tous il s'agit de la pauvreté, ne pouvoir satisfaire ses propres besoins et ceux de sa famille. Cette forme de maladie est celle qui porte atteinte le plus à notre qualité de vie. Car tu n'as pas les moyens tu ne manges ni en quantité ni qualité, tu ne dors pas assez, tout temps aussi tu es nerveux donc tous les maux que nous avons évoqués jusque là se retrouve dans le fait de vivre l'indigence. Et nous tous qui sommes là savons en toutes sincérité que par rapport à notre de vie nous n'avons pas encore de bonne avec cet élevage que nous vivons.

E: Donc toi tu penses que pour une bonne vie c'est de renoncer à l'élevage.

R1: Même si on ne renonce pas je suis sure les autres aussi peuvent le témoigner c'est très pénible de vivre de l'élevage. Pire aujourd'hui l'élevage traverse des moments très difficiles où il n'y a absolument rien. L'élevage est certes notre activité principale mais il ne permet pas satisfaire à nos besoins même ceux les plus simples comme manger bien et se reposer. Une bonne vie c'est une vie où on a des besoins qui sont couverts largement qui ne dépendent pas de l'élevage direct et sils parviennent à avoir d'autres revenus.

R 8 : Moi une bonne vie c'est la santé. ce que j'ajoute aussi c'est d'avoir beaucoup d'argent, avoir de quoi nourrir sa famille et avoir des matériaux.

E: donc pour lui avoir une bonne vie c'est d'avoir des facteurs de production qui sont un facteur principal de l'élevage entendue au sens de bœuf et de cheptel de petit ruminants, donc qui permet de satisfaire les besoins et celle d'à la famille.

E: est-ce que vous avez quelques choses à ajouter ou-bien vous êtes d'accord sur ce qui est une bonne vie. Quant est ce qu'on peut dire qu'une personne n'a pas une bonne vie?

Emmanuel : bon là y'a des choses qu'on doit relancer qui n'ont pas de rapport avec ce qu'on a demandé. Si tu pouvais leur dire que sur ce qu'ils ont évoqués tout à l'heure d'abandonner l'élevage, n'est-ce-pas, donc la question tu peux leur demandé si, est ce que vous pensez que il est nécessaire d'abandonner l'élevage selon vous pour avoir une meilleure qualité de vie?

R 1: Nous ne pouvons pas abandonner l'élevage parce-que nous n'avons pas d'autres choses à faire.

E : même si ce n'est pas d'abandonner totalement l'élevage, vous avez dit tout à l'heure que pour une bonne vie c'est pas de faire l'élevage, c'est ça n'est-ce-pas ?

R 3 : Oui! Ce n'est même pas mauvais de faire de l'élevage. Mais si cela nous fatigue plus qu'il nous sert aussi. Les conditions dans lesquelles nous menons l'élevage sont difficiles et cela ne nous permet pas de satisfaire nos besoins. Celui qui tente de satisfaire tous ses besoins avec le troupeau. Il ne s'en sortira pas.

Em: Oui, mais qu'est-ce-que pour les autres, il est nécessaire d'abandonner ou de diminuer ou-bien d'ajouter autres choses dans l'élevage.

E: Les autres que pensez-vous de ce qu'il dit?

R 10 : Lui ce qu'il a évoqué dans l'élevage ce n'est pas ce qu'on va abandonner parce-que moi personnellement je peux dire que c'est le milieu qui nous détermine à le faire, on ne peut l'abandonner vraiment. C'est notre nature nous les peulhs depuis nos anciens, nos grands pères et nos pères n'ont connus que l'élevage. Parce-que moi j'ai fait un constat, si quelque commence à avoir de l'argent même s'il travail hors élevage, qui vient ici, il finira forcément à l'aire l'élevage quelque part ou dans son village.

E: il dit aussi dans la même perspective qu'il ne s'agit pas d'abandonner l'élevage, parce-que c'est le milieu même qui les impose à travailler ou à continuer à faire l'élevage. Il donne l'exemple des agents qui viennent travaillé ici , bien vraie qu'ils ne soient pas des peulhs, ils ne sont pas aussi des éleveurs, ils travaillent dans d'autres structures mais ils finiront à faire l'élevage à côté, mais c'est parce-que, c'est que c'est le milieu qui les impose, il donne son cas propre même s'il fait autre chose il va faire l'élevage à côté parce-que c'est une identité, c'est une de leur caractéristiques, donc même si économiquement c'est pas viable, ça fait partie de leur mode de vie.

E:Que pensez-vous alors de l'élevage et des changements dont vous faites allusion par rapport à l'exercice de cette activité?

R3: Les conditions sont très difficiles actuellement il ne pleut pas assez il n'y a pas de pâturages, les saisons sèches deviennent de plus en plus longues. Ces tous des changements négatifs qui font que la vie des pasteurs est très pénible. Malgré cela, l'élevage reste notre patrimoine car nous l'avons hérité de nos ancêtres.

R 1: les changements c'est la sécheresse qui porte préjudice à notre activité et à notre mode de vie. Je pense qu'il faut changer de méthode car le processus s'est déclenché et c'est de pire en pire depuis la grande sécheresse dont tout le monde a entendu parler des dégâts depuis lors l'élevage n'a pas pu s'en remettre de ce choc.

E: Selon vous quelle est la solution face à ces changements dont vous parlez?

R 5: La solution ne serait certainement pas d'abandonner l'élevage, c'est impossible c'est équivaut à renier ce que nous sommes et qui nous fait vivre ici. Le peul est fait pour l'élevage alors il n'existe sans cet élevage. Il est peut être possible de changer si nous avons de l'aide.

R 3: La solution c'est de moderniser l'activité en aidant les éleveurs à avoir des animaux plus productifs s'il le faut réduire les effectifs. Toutes les autres activités les éleveurs les font par rapport à l'élevage donc la solution est de nous aider à surmonter les difficultés tout en continuant à faire l'élevage.

R 9: je pense que l'on peut le changer en faisant de «l'élevage» c'est à dire intensification, c'est-à-dire apporter les races nouvelles, des vaches laitiers, en tout cas changer de ce côté là au lieu de changer fondamentalement ou-bien même d'abandonner. Avec cette modernisation on sera plus productif et moins fatigué car imagine quand tu es en transhumance tu travailles jour et nuit pour surveiller le troupeau tu ne te repose pas, tu ne manges pas c'est très pénible.

E: Les autres donner qu'est-ce que vous en dites surtout toi X tu n'as pas répondu?

R 9: Comme ils l'ont dit vu que nous parlons de bonne vie, celle des éleveurs n'est pas des plus aisée car les conditions d'exercice de cette activité deviennent de plus en plus difficile. Par rapport aux changements tout ce qu'il faut faire c'est….

Em: Moi je pense que ce qu'il faut demander est ce que l'élevage dont ils vivent ça va comme ça ou il y a des choses à changer dans l'élevage pour accéder à une meilleure qualité de vie?

E: OK, maintenant, la manière dont vous vivez aujourd'hui, est ce que vous le souhaitez ainsi ou-bien vous aspirez à d'autres choses pour avoir une meilleure qualité de vie ?

R 5: Il y a beaucoup de choses qui nous manque surtout par rapport à la formation et à l'éducation mais aussi un manque de moyens et de projets qui viennent appuyer l'élevage dans son ensemble. Quand tu es instruit cela te permet de mieux gérer ta vie et tes activités. Ce manque d'éducation fait partie des choses qui nous bloquent maintenant. C'est en train de changer avec les jeunes générations mais avec nous ce n'est plus possible. Même pour cette modernisation de l'élevage dont on parle ce serait plus facile si nous étions instruits on pourrait

avoir une meilleure gestion de nos richesses pourquoi pas même réinvestir sur autre chose pour sortir de cet élevage qui est en survie.

R 3 : Moi je suis né et j'ai grandi ici en voyant les gens faire de l'élevage en même j'étais à l'école mais il y a beaucoup de choses qui ne me conviennent pas dans la manière dont nous faisons l'élevage. Économiquement cela pose problème car quand tu vois le cheptel qui passe par la chaque jour et la qualité de vie des populations sur place on se pose des questions sur la viabilité économique de ce système. Mais on ne peut que constater car il est très difficile de faire changer d'habitudes à nos parents qui sont là. Je pense qu'il faut renforcer l'éducation peut être d'ici les générations à venir les choses vont changer et les peuls commenceront à faire un élevage qui pourra permettent de satisfaire leurs besoins et qu'ils auront une qualité de vie meilleure.

E : les autres, qu'est-ce-que vous souhaitez changer dans votre vie pour avoir une meilleure qualité de vie ?

R 4: L'éducation est un problème. On continue de reproduire les mêmes problèmes que nos parents et nos ancêtres alors que les conditions ont beaucoup évolué. Au temps les conditions s'y prêtaient il y avait beaucoup de pluie et assez de pâturage et les besoins des populations correspondent aux retombés de l'élevage. Les ancêtres n'avaient aucune culture entrepreneuriale mais s'il vend un bœuf il pouvait avoir beaucoup d'argent qu'ils dépensent sans se soucier des possibilités d'investissement. Tout cela s'explique par le manque d'éducation et d'éveil c'est pourquoi nous pensons que c'est l'éducation qui va régler beaucoup de choses ici. Dieu merci, c'est en train de changer parce-que les jeunes sont plus conscient aujourd'hui, avant ils étaient moins instruits. Ce qui manque aussi c'est l'investissement parce que quelqu'un peut vendre ses bêtes et se retrouve avec 1.000.000 F CFA par exemple, il peut les gaspiller avec des choses de moindres valeurs.

E: les autres, qu'en pensez-vous?

R 6 : Je souhaiterais vraiment faire des études. Je sais que si j'avais des études ça pouvait contribuer considérable ou-bien changer mon mode vie ou-bien pour avoir une vie meilleure.

Emmanuel : est ce que leurs grands parents étaient insatisfaits comme eux ou est ce que pour eux ils étaient contents de leur vie comme ils étaient, parce-que on a l'impression que pour eux l'élevage ne peut pas satisfaire leur besoins sur le plan éducatif, sur le plan économique, éducatif et économique, ça ne les plaisent pas beaucoup. Est ce que leurs parents avaient les même besoins, est ce qu'ils avaient les mêmes satisfactions, est ce que c'était supportable de vivre comme ça ou-bien ?

R 10: Vous savez avant par exemple les équipements ne comptaient pas beaucoup pour les parents, ils avaient une vie plus simple que la nôtre mais aujourd'hui les choses ont changé et la demande en moyens est de plus en plus grande. Si tu regardes bien, tout le monde ici fait de l'élevage, n'empêche la qualité de vie dans toute zone n'est pas la meilleure on peine à avoir même une bonne alimentation c'est parce que l'élevage génère des revenus dérisoires. Les parents se sont rendus compte de l'importance des études, de l'importance de l'entrepreneuriat, mais ces leçons là ils les ont eues à partir des limites de l'élevage en tant que tel. Donc ils ont rendus compte qu'ils ne veulent plus vivre ce mode de vie là ou ce mode de vie là n'est plus à l'heure, parce-que jadis quand tu avais 200 bœufs, 300 bœufs c'était prestigieux aux yeux de toute la communauté, mais maintenant cela a changé. Il ne suffit plus d'avoir un cheptel, le compter et le regarder comme ça, mais c'est de peut être, en plus de cela ou-bien, le mode d'évaluation en tout cas de leur richesse a changé.

E: Est-ce que vos parents qui avaient 200 à 300 bœufs avaient une qualité de vie meilleure que la vôtre?

R 5: Avant quand tu avais 300 boeufs tu peux régler tous les besoins, et tu étais même considéré par tout le village (rire). Tu étais notable et rien ne se faisait sans que tu ne sois au courant.

R 7: Auparavant quand un enfant est né on lui donne comme premier cadeau des animaux essentiellement des vaches quand il grandit on lui montre son petit cheptel et on l'apprend à en prendre en charge. Dès le bas âge on est en train de le construire dans la pratique de l'élevage. Maintenant je donne 3 exemples si j'ai 300 bœufs ou-bien 200 bœufs, c'était en 73, c'est mon père qui me l'avait dit, il a dit qu'il avait un vieux qui avait plus de 300 boeufs et aujourd'hui il n'a rien, il a perdu presque la moitié des boeufs dans les fortes pluies de 2001. Avec le feu de brousse 1996 je suis témoin oculaire avait détruit beaucoup de chose ici. Les feux de brousse, la

sécheresse tout ça, c'est des faits naturels; ce n'est pas nous mais ce n'est pas bon pour nous. Mais maintenant, les choses ont changés parce-que tu pars à Dakar tu vois là-bas une personne qui habite ici, il a sa famille et sa femme, sa femme fait des enfants par exemple tu les amène les enfants à l'école, automatiquement tu constates une vie différence par rapport à ceux qui vivent ici. Car les aspirations ne sont pas les mêmes.

Em : est-ce que tous ici, ils voudraient avoir une vie citadine ?

E: souhaitez-vous vivre une vie citadine ou-bien une vie comme vos parents?

R3: Moi je préfère vivre comme la vie de mes ancêtres, n'empêche qu'il y a des changements à apporter dans notre mode vie. Vivre de l'élevage tout en étant à l'école et construire un avenir, avoir moyens de moins d'animaux et produire en qualité avec des revenus qui nous permettent de satisfaire nos besoins.

E: Les autres qu'en pensez-vous par rapport ce que nous venons de dire?

R 1: Personnellement la vie citadine ne m'intéresse pas trop. Si je parviens à tout avoir je préfère rester ici. Le mieux c'est d'essayer d'avoir toutes les commodités sur place et de rester sur place en améliorant nos conditions de vie. De mon point de vu, ce qui nous manque vraiment c'est de l'eau, parce-que nous parcourons plus de 10 KM pour avoir de l'eau. Vous savez aussi si l'eau était très proche ça va permettre de faire facilement notre travail et les enfants d'aller à l'école. Même si nous voulons faire autre chose on ne peut pas le faire, parce-que nous parcourons des distances importants toute la journée.

R 9: Je pense que l'on peut rester ici et avoir une bonne vie. Car il y a ceux qui vivent à Dakar et en ville dans une indigence totale par ce qu'ils gagent des miettes. Nous au moins quand on vend un petit ruminant on parvient à satisfaire un besoin et quand on vend une vache on gagne de l'argent. Ce qui nous manque c'est que nos conditions de vie soient améliorées que l'on puisse vivre de nos troupeaux en ayant des projets qui permettent à ce mode d'élevage de survivre. nous aussi devons faire des efforts en étant dans les dispositions d'être aidé en adoptant les pratiques modernes d'élevage avec un effectif réduit et productif. Si tout cela est fait ce sont les gens des villes qui viendront nous trouver ici car il fera bon vivre.

R 8 : Ce n'est pas parce que nous sommes des éleveurs que les autres sont plus riches que nous. l'élevage est très particulier par exemple ici tout le monde à un troupeau quelle que soit la taille et continu de vivre. Mais imaginer si chacun avait une boutique; il n'y aurait pas de client qui viendra acheter. Nous sommes riches de notre élevage nous avons besoin de changer un peu de manière de faire et là je pense que nous sommes contraints par les changements climatiques pas de pluies, les saisons courtes, pas de pâturages, et une brousse de plus en plus habitée. Il nous faudra s'adapter avec de nouvelles pratiques pour s'en sortir.

E : Merci beaucoup, ça était génial de partager avec vous cette discussion, merci encore une fois.

Focus groupe des femmes âgées de 25 à 35 ans

Widou Centre

E : Qu'est-ce qu'une bonne vie selon vous ?

R1 : Une bonne vie c'est celle d'une personne qui la santé mais aussi qui est instruite pour pouvoir prendre en charge sa vie. La différence entre notre vie et celle de nos ancêtres c'est surtout l'éveil et la scolarisation. Pour moi une vie sans éducation (école) ne peut pas être qualifié de bonne vie car c'est comme si tu étais aveugle et que tu tâtonnais pour retrouver le bon chemin. Par contre notre avantage aujourd'hui les générations récentes pour la plupart savent lire et écrire et cela améliore qualitativement notre vie. Je pense aussi que nos enfants auront une meilleure vie que nous car de plus en plus les enfants vont à l'école et avec cette éducation beaucoup de changements positifs auront lieu. Beaucoup de pratiques que nous continuons à faire sont des résultantes de notre ignorance c'est pourquoi je pense qu'après la santé c'est la scolarisation qui constitue le second pilier le plus important d'une bonne vie. Car quand une personne est bien éduquée et instruite elle pourra préserver sa santé en faisant attention à ce qu'il consomme, elle pourra aussi gérer ses biens économiques en prenant des décisions très sages. Alors elle aura une bonne vie.

R2 : Une bonne vie pour moi il faut avoir la santé et pouvoir aussi préserver cette santé. Tout le reste vient ou s'obtient avec la santé. Il faut dire qu'aujourd'hui il est très difficile d'avoir cette santé car nous avons un mode d'alimentation qui n'est pas saint du tout comparé à celui de nos ancêtres. Il y a beaucoup de maladie qui n'existait pas. Notre vie est différente de celle de ces ancêtres. Auparavant, il n'y avait pas du riz, et autres produits industriels ils vivaient du mil qui provenait de l'agriculture et des sous-produits de l'élevage. Ils savaient une meilleure santé que la nôtre. C'est bien l'école et autres mais je pense que celui qui n'a pas une bonne santé ne pourra pas aller à l'école. La base d'une bonne vie c'est la santé.

Les autres vous pouvez intervenir et donner vos avis par rapport à cette question. Selon vous quels sont les critères qui définissent une bonne vie ?

R3: Comme les autres l'ont dit la santé est la base de la bonne vie. Mais ce n'est pas tout car quand tu n'as pas une alimentation et que tu es trop fatigué aussi tu finiras par tomber malade. Je pense qu'il faut avoir les moyens de subvenir à ses besoins. Nous avons beaucoup d'avantages par rapport à nos grands-pères qui étaient très fatigués ils marchaient beaucoup

pour chercher de l'eau, lors des longues transhumances et voyageaient même à pieds. Nous avons plus de repos qu'eux l'eau est à coté et il y a des moyens de transport.

E: D'accord, pour vous une bonne vie, il faut une bonne santé, l'éducation, la bonne alimentation et avoir du repos. Les autres qu'est-ce que vous voulez y ajouter ?

R4: Une bonne vie, c'est aussi avoir de la civilisation (modernité) c'est-à-dire avoir toutes les commodités l'eau, l'électricité, le réseau (téléphone) pour vivre au même rythme que les autres. Alors que nos aïeuls vivaient sans tout cela c'est pourquoi ils ne savaient rien de ce qui se passe dans le monde. Les femmes aussi étaient très fatiguées elles allaient aux champs, pilaient le mil, cherchaient l'eau, s'occupaient du troupeau. Elles n'avaient presque pas de vie. Aujourd'hui, tous ces problèmes sont en train d'être réglé nous avons plus de repos, des machines pour piler (moulin), il n'y a plus de champs, l'eau est à proximité.

E : Tu as dit une bonne vie c'est la mondialisation, la civilisation et ils ne connaissaient pas la civilisation et ils étaient des analphabètes et est-ce que tu peux nous expliquer ces termes « mondialisation », « civilisation» et « analphabètes » pour ceux qui ne comprennent pas le français pour plus d'explication afin de mieux animer la discussion.

R4: Le mot analphabète signifie ceux qui n'ont pas fait les bancs, la civilisation c'est la propriété, l'hygiène, la qualité de ce que nous mangeons, ce que nous portons être comme les autres qui sont en ville ou bien même en Europe, les Etats Unis etc.

E : Selon comment faire pour avoir une bonne vie ?

R6: pour avoir une bonne vie il faut éduquer les enfants, car avec la scolarisation beaucoup de choses peuvent changer. Quand les enfants vont réussir ils vont aider leur parents s'ils ont un bon travail aussi ils pourront aider toute la localité en amenant des projets qui aident la population.

R1: pour avoir une bonne vie, nous devons essayer de voir ce qui manquait à nos ancêtres et essayer de l'avoir. En premier l'éducation c'est elle seulement qui peut changer les choses. Car l'éducation permet à l'enfant d'être éveillé, avoir une ouverture d'esprit. Par exemple si nos enfants sont scolariser jusqu'à avoir un très haut niveau ils pourront améliorer l'élevage en le modernisant. L'élevage doit être changé dans la manière dont nos grands-pères et nos pères le faisaient. L'époque où on doit continuer à suivre le troupeau sans vivre aisément est révolue c'est l'éducation des enfants qui peut changer les choses en modernisant l'activité pour que les pasteurs puissent y tirer profit. A côté de cette scolarisation de nos enfants aussi il faut les initier

à l'élevage car c'est pour la préservation de notre culture qu'il ne faut jamais abandonner définitivement.

E : c'est intéressant car depuis le début de la discussion vous avez parlé de la bonne vie en évoquant les autres aspects tels que l'éducation, la bonne alimentation etc. alors qu'ici nous sommes dans une zone d'élevage. C'est X qui vient d'en parler en insistant sur le fait que l'élevage est difficile mais à ne pas abandonner tout de même. Vous avez aussi évoqué plusieurs changements en cours. Comment est-ce que vous articuler tout cela avec l'élevage qui constitue l'activité principale et vos critères de définition de la bonne vie ?

R4 : Il y a des changements l'élevage en tant que tel est difficile mais la manière dont la pratiquons aujourd'hui est quand même moins pénible par comparaison à nos ancêtres. Car nous avons l'eau, pendant la transhumance aussi la brousse est maintenant habitée, il y a des villages et des marchés où on peut avoir beaucoup de choses pour satisfaire nos besoins. Les femmes, les veilles personnes et les enfants ne transhument pas ils restent sur place ce qu'ils leur procurent un repos alors jadis tout le monde partait avec le troupeau, les femmes enceintes et même celles qui venaient de donner naissance ne pouvaient rester car en saison sèche personne ne restait sur place et il n'y avait pas beaucoup d'habitants dans les villages. Donc c'est beaucoup de changements positifs surtout dans la manière dont l'élevage est pratiquée aujourd'hui qui même s'il y a toujours des difficultés c'est moins qu'avant.

R3: Il y a beaucoup de changements qui touchent à l'élevage et notre mode vie. Par exemple par rapport à l'eau on pouvait rester toute une journée ou bien même des jours sans en avoir. Celui qui n'a pas de l'eau de boisson ne pense pas à se laver ou à faire le linge donc il n'y pas hygiène du tout ce qui peut favoriser des maladies. Par rapport à l'alimentation, elle était pauvre car les produits n'étaient pas variés et dès fois il n'était même pas possible d'en avoir parce qu'il y'avait même pas de marchés et peu de commerçants.

Je pense aussi pour une bonne vie les gens doivent habiter ensemble en créant de grands villages au lieu d'avoir de petits campements qui parfois c'est un seul ménage. En habitant ensemble les petits campements vont disparaitre et il y aura plus d'espace dans la brousse pour les pâturages. Et si les gens vivent ensemble, il y aura une solidarité et entre aide.

R1:Comme elle a l'a dit seulement. Tu sais que les peulhs vivent en famille mais ils ne cohabitent pas ensemble. Tu viens dans cette zone ces gens habitent par ici et d'autres là-bas. Même s'il y a lieu qu'on les aide comme la construction des écoles ; tu sais aussi : une seule famille qui se considère comme un village ne peut pas avoir une école pour faire apprendre ses

quelque chose à ses enfants. Mais s'il y a un rassemblement entre eux par la cohabitation, ils peuvent avoir tout ce qu'ils veulent. Ils peuvent avoir des écoles, faire des jardins, tout ça ils peuvent avoir et tout. En effet, s'ils ne sont pas unis ; ils ne peuvent pas bénéficier des projets ou bien des aides de l'Etat. S'ils n'habitent pas ensemble c'est parce que chacun poursuit son troupeau qui a besoin d'espace mais pour une bonne vie aussi ils doivent habiter ensemble créer de grands villages qui seront très animé (ambiance) et il y'aura tout pour vivre aisément, de grands marchés, des écoles avec tous les niveaux (primaire, moyen, secondaire), des dispensaires, comme dans les grandes villes comme Daara.

E : Vous dites que la dispersion des habitations est un problème. Est-il possible que les peuls habitent ensemble en créant de grands villages tout en continuant à garder le grand cheptel ?

R1: Pour moi ce serait difficile au début mais c'est possible à conditions qu'ils soient sensibilisés sur le fait qu'en habitant ensemble ils peuvent y tirer beaucoup de profit des aides et autres projets de développement. Cela va aussi libérer la brousse pour permettre aux troupeaux d'avoir plus d'espaces de pâturages.

R7: Comme l'a dit celle qui m'a précédé sans habiter ensemble les peuls sont très dispersés cela ne joue plus en leur faveur car on a besoin de plus des projets et des aides. De plus la brousse se rétrécit d'où l'importance d'habituer ensemble qui permet de créer de grands villages où on peut tout avoir, de pouvoir bénéficier des projets mais aussi de libérer la brousse pour en faire des espaces de pâturages.

E : Nous avons échangé avec les personnes âgées qui ont affirmé qu'il y 'avait plus de solidarité et entre aide au paravent. Vous vous dites que la dispersion des habitations constitue un handicap et qu'il y aurait plus de solidarité quand les gens habitent ensemble. Existent-ils d'autres changements que vous avez observés dans les modes de vie?

R4: Il y a beaucoup de changements, notre vie actuelle est très compliquée car nous avons beaucoup de besoins surtout nous les femmes. Jadis une femme pouvait avoir juste un seul bon boubou qu'elle porte dans les cérémonies sans problème. Mais actuellement les femmes vont dans beaucoup de fêtes et elles préfèrent avoir une nouvelle tenue à chaque instant. Pour la cuisine nous cuisinons que du riz et d'autres nouveaux produits qui n'existaient pas avant. Avec ces changements nous avons une vie plus aisée mais elle est aussi plus compliquée que celle de nos ancêtres.

E: Si je comprends actuellement il y a beaucoup de nécessités ce qui entraine une réorganisation de la famille et la gestion du troupeau.

R6: Oui nous avons beaucoup de besoins mais il y a aussi le fait que rien n'est produit ici on acheté tout. Quand tu achètes tout tu as l'impression que tu dépenses beaucoup mais on est obligé car aucune des dépenses ne peut vraiment manquer. Cette situation fait que les gens sont devenus plus calculateurs même au sein des familles où chacun cherche à s'en sortir. Ce n'est pas qu'il y a moins de solidarité mais c'est que les gens ne peuvent pas aider car tout le monde survie. Si tu vois des frères se séparent pour que chacun cuisine à coté c'est parce que chacun a sa petite famille (épouse et enfants) dont il doit assurer les besoins.

E: Qu'est ce qui a amené tous ces besoins nouveaux est ce que ce sont des nécessités?

R3: comme on l'a expliqué il y a beaucoup de changements qui touchent même à nos besoins vitaux. Nous avons besoins de nourriture, d'habits, d'équipements, de santé, on paie l'eau, on paie les aliments de bétails. On achète tout pour résumer donc c'est normal que les gens deviennent de plus en calculateur et quand tu calcule tu n'aides personnes car tu penses qu'en aidant quelqu'un tu perds ce que tu as.

R6: Cela nous inquiète aussi. Parce que ce qu'il existe une pression sur les ressources pâturage il faut limiter le bétail car les ressources actuelles ne peuvent faire vivre les troupeaux qui sont là. Ce n'est pas le refus de donner aux autres prochains. C'est juste ça. Tu sais que les anciens n'étaient pas éveillés et ils ne savaient rien en possédant beaucoup de ressources mais nous voyons un seul pantalon, chez la femme aussi un seul boubou. Ils ne vivaient que du mil ne mangeaient pas ce que nous mangeons actuellement et ils ne voyageaient pas en voiture. Donc, actuellement, c'est ce qui a poussé à certains d'avoir ce comportement car nous avons beaucoup de besoin.

R 6: Au temps il y avait des champs de mils et aujourd'hui il n y a pas ça. De nos jours rien n'est produit ici. c'est le riz qui constitue notre principal produit d'alimentation de base et on l'achète le sac de 50 Kg ou bien en achetant par détail.

E : Comme vous avez dits avec ces changements vous avez votre cheptel et vous abandonner l'élevage en laissant les gens les jeunes d'apprendre comme vous aviez dits des analphabètes pour les faire s'inscrire dans l'école comme vous l'avez dit. Et vous si vous avez appris comment vous allez vivre ? C'est inquiétant ou c'est bien, comment voyez-vous cela? Surtout vos les femmes et votre vie s'est différente de vos parents.

R 6: Pour moi il n'y a pas d'inquiet mais les enfants qui viendront, il faut qu'ils aient apprendre soit l'école ou le coran. Parce que s'ils apprennent, ils auront des bagages intellectuels ou religieux pour la génération future. Et si tu n'apprends pas tu ne vas pas participer dans le monde du futur. Cause pour il faut qu'ils apprennent le français ou le coran. Moi c'est ça que je vois

E : Que pensez-vous de son point de vue ?

Notre société est en pleine transformation sur beaucoup de plans. Par exemple pour l'élevage on voit qu'il pleut de moins en moins, la brousse aussi est habitée et le bétail n'a plus assez d'espace pour le pâturage. Ces changements qui touchent l'élevage a des conséquences sur les pasteurs eux même. Pour l'alimentation il y a le passage du mil au riz et donc pour les générations futures il y aura d'autre chose qui va remplacer le riz peut être. Il y a aussi beaucoup de maladies qui nos ancêtres ne connaissaient pas. Avec autant de problème la solution c'est de diminuer le cheptel essayer d'avoir d'autres activités économiques. Car il y a beaucoup de besoins et l'élevage ne permet plus de satisfaire à tous ces besoins.

E: Que pensez-vous de ce qu'elle dit? Est ce qu'il est possible de diminuer le cheptel?

R8: On change l'élevage en diminuant le bétail. Il faut avoir un effectif raisonnable facilement maitrisable c'est à dire que l'on peut entretenir pour qu'il soit rentable. Le problème ici c'est qu'il y a des éleveurs qui ont de grands effectifs et ils sont les plus fatigués et quand tu les regardes pour certains ils sont les plus pauvres car ils ne se reposent pas ils ne mangent pas correctement et ils portent des haillons. Tout cela parce que l'entretien du troupeau leur pose problème. Mais c'est cela le problème car l'élevage est une pratique culturelle personne ne veut diminuer son troupeau même s'ils savent que c'est vraiment dure la vie actuellement avec un grand cheptel. Je pense que la solution c'est d'aménager des jardins pour les femmes, elles vont arrêter de transhumer avec le troupeau, elles vont travailler ce qui les permet de gagner quelque chose et suivre l'éducation des enfants. Je pense que cette solution aux femmes d'avoir des revenus en dehors du bétail et les enfants iront à l'école. Car nous avons remarqué qu'à cause des transhumances certains enfants surtout des hameaux abandonnent leurs études.

R5: Moi je vois que l'élevage va disparaître car les conditions se détériorent de plus en plus chaque année les pluies diminuent et les pâturages deviennent plus rares. Même avec la transhumance les éleveurs ne savent plus où y aller car c'est à peu près la même chose. C'est pourquoi on voit maintenant des gens qui ont des maisons à Dahra ou bien même dans les autres villes pour la location ou bien les éleveurs riches qui paient l'école pour leurs enfants tout cela

n'existait pas il y a quelque années. Je pense que certains éleveurs perçoivent les menaces sur l'élevage et ils anticipent.

R3: Je suis d'accord avec elle sur certains points que les choses sont en train de changer. Avec le problème des pâturages et de la pluie les pasteurs se rendent compte qu'il faut changer. Mais les peuls ne vont jamais abandonner l'élevage cela c'est sure car c'est aussi notre culture. Avec l'école et les migrations des gens qui vont en ville et reviennent on voit des changements positifs surtout par rapport à comment nos ancêtres vivaient. Tu remarques qu'il y a des constructions en dur comme dans les villes et quelqu'un qui construit en dur cela limite les transhumances et permet aux enfants d'être scolariser.

E: En quoi l'éducation joue un rôle dans la qualité d'une meilleure vie?

R1: L'école est très importante parce ce qu'ici jusqu'à récemment la scolarisation n'était pas trop développée. Les populations sont très conservatrices et les parents socialisent les enfants qu'à l'élevage. C'est pourquoi quand ils grandissent ils perpétuent cette tradition quelques soient les difficultés. Mais quand on a la chance de faire l'école on change plus rapidement de vision. C'est pourquoi nous pensons qu'avec l'école les choses vont changer car les gens deviennent de plus en plus éveillés. L'école permet aux enfants d'avoir des connaissances dans d'autres domaines.

E: selon vous comment l'élevage doit s'adapter pour faire aux difficultés que vous avez évoqué ?

R2: Pour s'adapter aux conditions actuelles, les éleveurs doivent limiter le cheptel, en essayant d'avoir un effectif réduit et productif. Il faut aussi que les éleveurs s'investissent dans d'autres activités par exemple ouvrir des boutiques dans les villages.

R 1: Pour aider les éleveurs à s'adapter il faut qu'il y ait beaucoup de projets dans zone pour employer les populations. Ainsi les éleveurs auront d'autres sources de revenus que le troupeau.

R 8: Il faut que l'on fasse un élevage moderne où les animaux seront réduit et ils seront nourris et entretenus sur place. Et vu que nos races ne sont pas adaptées à ce mode d'élevage il faut chercher des plus productrices par exemple des vaches qui produisent beaucoup de lait cela peut permettre aux femmes d'avoir du lait pendant toute l'année et pourquoi pas installer des laiteries dans la zone.

Vous faites référence à la vie de vos ancêtres selon vous qu'est ce qui vous distinguent?

R7: Il y a beaucoup de choses qui nous distinguent. Nous avons plus d'avantage que nos ancêtres car l'eau était très rare car il y'avaient peu de forage avec des distances très longues. Sur le plan des moyens de déplacement je peux dire qu'il n'y avait pas de véhicule, les populations marchaient beaucoup; certains n'ont jamais mis les pieds à Dakar ou dans d'autres villes. Mais ils avaient une meilleure santé que nous car ils ne connaissaient pas toutes les maladies que nous vivons aujourd'hui par exemple la tension, le diabète. Ces maladies sont dues aux transformations de notre alimentation on ne mange plus de mil, il n'y a plus de lait de qualité car les pâturages ne sont plus de qualité. Nous vivons essentiellement de riz et des produits qui viennent d'ailleurs. c'est pourquoi nous sommes malades. On pense que nous avons une meilleure vie que nos ancêtres mais en réalité c'est que nous avons plus de confort mais ils vivaient mieux que nous car ils étaient en bonne santé. Parce qu'ils mangeaient très saint sans trop de produits artificiels.

R3: nous avons une meilleure vie que nos grands-mères qui faisaient des activités pénibles aller aux champs, piler le mil, chercher de l'eau sur de très longues distances. Nous aujourd'hui on pile très rarement car on ne cultive plus ici et même si on achète du mil nous avons un moulin ou bien achète des produits déjà transformés. En tout cas nous sommes moins fatiguées que nos mamans et de grand-mères.

R1: Attention je suis pas d'accord que nos ancêtres étaient plus fatigués que nous. Nous sommes plus fatiguées car nous avons plus de préoccupations et de besoins sur ce plan nous plus fatiguées. On nous raconte que jadis tu voyais une dame qui avait qu'une seule bonne tenue qu'elle porte dans toutes les cérémonies sans problème. Nous toutes qui sommes là nous avons des valises remplient de tenues mais nous continuons d'en chercher. Il y a aussi le fait que nos ancêtres s'entre-aidaient plus que nous aujourd'hui il n y a presque pas de cohésion sociale et de solidarité entre les populations.

E: Qu'est ce qui est plus dynamique et plus active entre vous et vos ancêtres?

R4: Nos ancêtres étaient plus actives et plus fatigués que nous. Nous actuellement nous vivons dans un confort car il y a beaucoup de forages l'eau n'est plus un casse-tête, on se déplace facilement avec les voitures pour les longues distances et les charrettes entre les villages. Alors que nos ancêtres n'avaient pas tout cela ils marchaient des kilomètres pour chercher de l'eau. Par exemple les commerçants de bétails conduisaient le troupeau à pieds jusqu'au marché de Dahra.

R3: Comparé à nos ancêtres nous ne sommes fatigués. On l'a dit tantôt les femmes cultivaient aux champs, pilent le mil, traient les vaches, collectent du bois de chauffe, et prennent soins aux enfants. Nous actuellement nous ne pouvons pas faire tout cela nos organismes ne peuvent même pas le supporter.

E: c'est nous qui vous remercions d'avoir accepté de participer à cette discussion.
